# A subset of activated fibroblasts is associated with distant relapse in early luminal breast cancer

**DOI:** 10.1186/s13058-020-01311-9

**Published:** 2020-07-14

**Authors:** Claire Bonneau, Antoine Eliès, Yann Kieffer, Brigitte Bourachot, Sylvain Ladoire, Floriane Pelon, Delphine Hequet, Jean-Marc Guinebretière, Christophe Blanchet, Anne Vincent-Salomon, Roman Rouzier, Fatima Mechta-Grigoriou

**Affiliations:** 1 Stress and Cancer Laboratory, Equipe labelisée Ligue Nationale Contre le Cancer, Institut Curie, PSL Research University, 26, rue d’Ulm, F-75005 Paris, France; 2 Inserm U830, Institut Curie, PSL Research University, 26, rue d’Ulm, F-75005 Paris, France; 3 Department of Surgery, Institut Curie Hospital Group, 35 rue Dailly, 92210 Saint-Cloud, France; 4 Inserm U1231, Chemotherapy and immune response, Center Georges François Leclerc, 1 rue du Professeur Marion, 21000 Dijon, France; 5 Department of Pathology, Institut Curie Hospital Group, 35 rue Dailly, 92210 Saint-Cloud, France; 6 Department of Pathology, Institut Curie Hospital Group, 26, rue d’Ulm, 75248 Paris, France; 7 Inserm U900, Cancer et génome : bioinformatique, biostatistiques et épidémiologie, Institut Curie, 35 rue Dailly, 92210 Saint-Cloud, France; 8 UR 7285, Risques cliniques et sécurité en santé des femmes et en santé périnatale, Versailles Saint Quentin en Yvelines University, 2 avenue de la source de la Bièvre, 78180 Montigny-le-Bretonneux, France

**Keywords:** Luminal breast cancer, Metastases, Cancer-associated fibroblasts, Stroma, CDH11, Cadherin 11, Tumor infiltrating lymphocytes, TILs, Tumor micro-environment

## Abstract

**Background:**

Early luminal breast cancer (BC) represents 70% of newly diagnosed BC cases. Among them, small (under 2 cm) BC without lymph node metastasis (classified as T1N0) have been rarely studied, as their prognosis is generally favorable. Nevertheless, up to 5% of luminal T1N0 BC patients relapse with distant metastases that ultimately prove fatal. The aim of our work was to identify the mechanisms involved in metastatic recurrence in these patients.

**Methods:**

Our study addresses the role that autonomous and non-autonomous tumor cell features play with regard to distant recurrence in early luminal BC patients. We created a cohort of T1N0 luminal BC patients (tumors between 0.5–2 cm without lymph node metastasis) with metastatic recurrence (“cases”) and corresponding “controls” (without relapse) matched 1:1 on main prognostic factors: age, grade, and proliferation. We deciphered different characteristics of cancer cells and their tumor micro-environment (TME) by deep analyses using immunohistochemistry. We performed in vitro functional assays and highlighted a new mechanism of cooperation between cancer cells and one particular subset of cancer-associated fibroblasts (CAF).

**Results:**

We found that specific TME features are indicative of relapse in early luminal BC. Indeed, quantitative histological analyses reveal that “cases” are characterized by significant accumulation of a particular CAF subset (CAF-S1) and decrease in CD4^+^ T lymphocytes, without any other association with immune cells. In multivariate analysis, TME features, in particular CAF-S1 enrichment, remain significantly associated with recurrence, thereby demonstrating their clinical relevance. Finally, by performing functional analyses, we demonstrated that CAF-S1 pro-metastatic activity is mediated by the CDH11/osteoblast cadherin, consistent with bones being a major site of metastases in luminal BC patients.

**Conclusions:**

This study shows that distant recurrence in T1N0 BC is strongly associated with the presence of CAF-S1 fibroblasts. Moreover, we identify CDH11 as a key player in CAF-S1-mediated pro-metastatic activity. This is independent of tumor cells and represents a new prognostic factor. These results could assist clinicians in identifying luminal BC patients with high risk of relapse. Targeted therapies against CAF-S1 using anti-FAP antibody or CDH11-targeting compounds might help in preventing relapse for such patients with activated stroma.

## Background

Breast cancer (BC) is the most frequent cancer in women with an estimated 2.08 million new cancer cases diagnosed in 2018 (24.2% of all female cancers) [1]. BC is a heterogeneous disease with various prognoses. Among the four main BC subtypes (luminal A, luminal B, *basal-like* and human epidermal growth factor receptor 2 (HER2)-enriched [2–4]), luminal BC express hormonal receptors and are considered sensitive to endocrine therapy. They represent 70% of all BC and almost 85% of early BC [5–7]. The prognosis for early luminal BC patients is very good with a disease-free survival rate of 99.1% at 5 years for luminal T1N0 BC, i.e., small luminal tumors (smaller or equal to 2 cm) without lymph node metastasis [8]. Despite this overall good prognosis, up to 5% of patients relapse, even sometimes belatedly, and die from metastatic disease. Thus, the balance between overtreating most patients who will not relapse, and undertreating patients who will ultimately relapse, remains a challenging clinical problem [9–11]. Hence, only a few comprehensive studies have been published on luminal T1N0 BC. On the whole, high proliferation index (histological grade or Ki67), young age at diagnosis, progesterone receptor (PR) expression, and lymphovascular invasion are known prognostic factors in this subgroup of patients [10]. More recently, commercial classifications have been developed as prognostic and/or predictive tools. For example, the Prosigna™ test combines tumor size, lymph node status, and the transcriptomic PAM50 gene signature (as defined in [12]) to provide the molecular BC subtype, an individualized prognostic score called “ROR score” (risk of recurrence score) for predicting metastasis-free survival at 10 years [13, 14]. In a study focusing on T1N0 ER^+^/HER2^−^ BC, the ROR score was a better prognostic tool than histological grade and Ki67 [14]. Other studies have indicated that the ROR score is superior to a standardized immunohistochemical classification, but they did not focus on luminal T1N0 BC [13, 15]. It is important to note that until now there is no biological or translational study focusing on T1N0 luminal BC. Subsequently, there exists a real need for new strategies to determine the long term prognosis of these patients.

It is now well established that tumor microenvironment (TME) plays a key role in tumor development and progression. In addition to cancer cells themselves, various normal cells, such as cancer-associated fibroblasts (CAF), immune and endothelial cells, and pericytes, are embedded in the extracellular matrix (ECM) and are all involved in numerous phases of tumor growth and spread. Indeed, there is now long-established evidence for the role of vascular density, as well as innate and adaptive immunity, in tumorigenesis. Carcinoma-associated fibroblasts (CAF) constitute one of the most abundant stromal components in solid tumors [16–20]. Although CAF heterogeneity has been largely underestimated in the past, several recent studies have identified several CAF subsets [21–24]. In particular, the concomitant analysis of several stromal markers, including fibroblast activation protein (FAP), smooth-muscle α actin (SMA), and integrin β1 (CD29), demonstrated the existence of at least 4 different CAF sub-populations (named CAF-S1 to CAF-S4) in human BC [22, 23]. CAF-S1 (FAP^Hi^ CD29^Med^ αSMA^Hi^ PDGFRβ^Med-Hi^ FSP1^Low-Hi^) and CAF-S4 (FAP^Neg-Low^ CD29^Hi^ αSMA^Hi^ PDGFRβ^Low-Med^ FSP1^Low-Med^) are myofibroblasts that accumulate mainly in aggressive BC, i.e., HER2 and triple-negative (TN) BC [22, 23]. Both subpopulations defined either by ECM (CAF-S1) or perivascular/contractile (CAF-S4) signatures have been validated in other studies in distinct adenocarcinomas, as well as in mouse models [21, 24–29]. In addition, the first single cell data from human and mouse cancers confirmed the existence of these two myofibroblastic CAF subsets [30–32]. The two other CAF subpopulations, CAF-S2 (FAP^Neg^ CD29^Low^ αSMA^Neg^ PDGFRβ^Neg^ FSP1^Neg-Low^) and CAF-S3 (FAP^Neg^ CD29^Med^ αSMA^Neg-Low^ PDGFRβ^Med^ FSP1^Med-Hi^), are detected in tumors but also in healthy tissues, suggesting that they could be normal-like resident fibroblasts. Taken as a whole, these observations show that both CAF-S1 and CAF-S4 can be detected in distinct cancer types and species, thereby highlighting their relevance in cancer research. Interestingly, the CAF-S1 subset exhibits immunosuppressive properties by attracting CD4+CD25+ T lymphocytes, increasing their survival, enhancing their differentiation in regulatory T cells, and promoting their activity [22]. These data were previously confirmed in human BC observations inferred from pancreatic cancer mouse models and derived organoids [21–24, 33]. Finally, although CAF-S2 are mostly detected in luminal patients, some luminal patients accumulate either CAF-S1 or CAF-S4, suggesting that these patients could show distinct clinical features. Based on the lack of knowledge relating to relapse in luminal BC, the objective of the current study was to decipher the mechanisms involved in distant recurrence of early luminal BC. We investigated cancer cell-autonomous roles, together with the impact of TME, and took into account adaptive immunity, vascularization, and CAF subsets.

## Methods

### Patient cohort

Patient data were selected from the Institut Curie Hospital Group (IC) BC database. 57,665 patients with in situ or invasive BC treated at IC, at the Paris or Saint-Cloud sites (France), from 1980 to 2010 were considered for this case-control study. We selected 3739 female patients with unifocal invasive BC, T1b, or T1c N0M0, expressing estrogen receptor (ER ≥ 10%) without overexpression of HER2 (HER2 0, 1+, or 2+ without gene amplification in in situ hybridization, as defined by the American Society of Clinical Oncology/College of American Pathologists Clinical practice guidelines [34]). Patients had been treated at IC by primary surgery between 2003 and 2010 (see flow chart in Additional File 1: Fig. S1). Among this population, there were 52 patients with distant relapse (excluding local, contralateral, or axillary lymph node relapse)—then called “cases”—and available samples (Additional File 1: Fig. S1). We chose 52 patient” controls” from the same population with a 1:1 match in age, grade, and Ki67. Clinical, pathologic, and therapeutic characteristics of the cohort studied in this paper are described in Table 1.

**Table 1:** Patients characteristics

	Global population (***n*** = 104)	Controls (***n*** = 52)	Cases (***n*** = 52)	***p*** value
**Age at diagnosis**	55 [32; 86]	55.5 [32; 86]	54.5 [38; 82]	0.77^a^
**BMI (kg/m**^**2**^**)**	23.9 [17.3; 44.1]	24.1 [17.3; 44.1]	23.8 [19.1; 41.6]	0.96^a^
**Tumor size (in mm)**	15 [6; 20]	15 [6; 20]	15 [10; 20]	0.59^a^
T1b	11 (10.6%)	5 (9.6%)	6 (11.5%)	1^b^
T1c	93 (89.4%)	47 (90.4%)	46 (88.5%)	
**Histological subtype**				0.29^b^
N.S.T.	87 (83.6%)	42 (80.8%)	45 (86.5%)	
Lobular	11 (10.6%)	8 (15.4%)	3 (5.8%)	
Mixed	1 (1%)	0	1 (1.9%)	
Other	5 (4.8%)	2 (3.8%)	3 (5.8%)	
**Grade**				0.86^b^
I	23 (22.1%)	12 (23.1%)	11 (21.2%)	
II	57 (54.8%)	27 (51.9%)	30 (57.6%)	
III	24 (23.1%)	13 (25%)	11 (21.2%)	
**Neoplastic emboli**	33 (35.1%)	14 (31.1%)	19 (38.8%)	0.51^b^
**Intraductal carcinoma**	75 (72.1%)	35 (67.3%)	40 (76.9%)	0.38^b^
**ER (%)**	80 [14; 100]	80 [20; 100]	85 [14; 100]	0.64 ^a^
**PR (%)**	60 [0; 100]	65 [0; 100]	60 [0; 100]	0.33 ^a^
**HER2 score**				0.90^b^
0	73 (70.2%)	36 (69.2%)	37 (71.2%)	
+	22 (21.2%)	12 (23.1%)	10 (19.2%)	
++ ISH −	9 (8.7%)	4 (7.7%)	5 (9.6%)	
**Ki67 (%)**	19 [1; 70]	21 [1; 60]	16.5 [5; 70]	0.65 ^a^
**Mitotic index**	8.5 [0; 100]	8 [0; 100]	9.5 [1; 60]	0.78 ^a^
**Surgical treatment**				
Lumpectomy	95 (91.3%)	50 (96.2%)	45 (86.5%)	0.16^b^
Total mastectomy	9 (8.7%)	2 (3.8%)	7 (13.5%)	
Sentinel lymph node biopsy	53 (51%)	30 (57.7%)	23 (44.2%)	0.23^b^
Axillary lymphadenectomy	51 (49%)	22 (42.3%)	29 (55.8%)	
**Other treatments**				
Chemotherapy	23 (22.1%)	13 (25%)	10 (19.2%)	0.63^b^
Radiotherapy	91 (87.5%)	49 (94.2%)	42 (80.8%)	0.07^b^
Endocrine therapy	96 (92.3%)	48 (92.3%)	48 (92.3%)	1^b^
**Evolution**				
Local relapse	11 (10.6%)	0	11 (21.2%)	0.0005^b^
Controlateral relapse	3 (2.9%)	0	3 (5.8%)	0.24^b^
Axillary lymph node relapse	5 (4.8%)	0	5 (9.6%)	0.05^b^
Distant relapse	52 (50%)	0	52 (100%)	
Bone metastases	41 (39.4%)	0	41 (78.8%)	
Visceral metastases	24 (23%)	0	24 (46.2%)	
**Death**	31 (29.8%)	0	31 (59.6%)	< 0.0001^b^

^a^Mann-Whitney test to compare controls and cases

^b^Fisher exact test to compare controls and cases

*BMI* body mass index, *ER* estrogen receptor, *PR* progesterone receptor

### Ethics

All patients included in our study and treated at IC were informed by their referring oncologist that their biological samples could be used for research purposes. All patients signed informed consent of non-opposition for the collection of excessive tumor samples and molecular analysis. The projects developed here are based on surgical residues, available after histopathological analyses, and not required for diagnosis. There is no interference with clinical practice. Human experimental procedures for TME studies driven in Dr. F. Mechta-Grigoriou’s laboratory were approved by the Institutional Review Board and Ethics committee of the Institut Curie Hospital group (approval February 12, 2014) and CNIL (Commission Nationale de l’informatique et des Libertés) (N° approval: 1674356 delivered March 30, 2013).

### Immunohistochemistry

Patient samples were obtained from the Department of Pathology at IC. A pathologist selected a representative sample for each tumor. For the different staining performed in this study, the embedded tissues were sectioned with a maximum delay of 3 months. For each set of experiments (CAF subsets, immune and cancer cells), consecutive sections of paraffin-embedded BC tissues (3 μm) were performed and then stored in a cold room. Sections were promptly stained using a streptavidin-peroxidase protocol (Vectastain ABC kit; Vector Labs #PK-4000, or Dako EnVision Flex HRP #DM822) on Autostainer Labvision (Thermoscientific, N°LVE-LV1.3-0463) and Benchmark apparatus (Ventana) for staining of CAF and immune cells respectively. In brief, paraffin-embedded sections were incubated with specific antibodies (Additional File 2: Table S1 for antibody references and conditions) recognizing CAF subsets—FAP (fibroblast activation protein α1), αSMA (smooth muscle α-actin), integrin β1/CD29, S100-A4/FSP1 (fibroblast-specific protein 1), and PDGFRβ (platelet-derived growth factor receptor-β)—and lymphocytes (CD4, CD8, FOXP3, IL17, T-Bet, CD20), dendritic cells (DC LAMP), macrophages (CD163), PD-1, PD-L1, CD31, and CDH11. Incubation took place in Dako Wash Buffer (Dako, #GC807) for 1 h at room temperature following unmasking in either Tris/EDTA buffer, pH = 9 (Dako #S2367), or citrate buffer, pH = 6 (Dako #S2369) (depending on the primary antibody, Additional File 2: Table S1). Unmasking was carried out in a microwave for 20 min at 97 °C.

### Pathological and immunostaining analyses

All pathological analyses were performed after scanning slides with the Philips Ultra-Fast Scanner 1.6 RA, except for the immunological slides that were numerized with Nanozoomer HT2.0 (Hammamatsu) at × 20 magnification. Those analyses were performed blinded for clinicopathologic data. The percentage of stroma was evaluated on H&E (hematoxylin and eosin) sections. For microenvironment analysis, only the intra-tumoral stroma was considered. The adiposity of the stroma was evaluated using a semi-quantitative scoring: 0 when the adipose content of the stroma was less than 1% of the surface, 1 between 1 and 10%, 2 between 11 and 30%, and 3 if superior to 30%.

For staining of each CAF marker, CDH11 and E-cadherin (CDH1), histological scoring (H score) was performed considering all tumor fields observed on sections and given as a function of percentage of stained cells (considering fibroblasts for CAF markers, cancer cells for CDH1 and both CAF and cancer cells analyzed separately for CDH11) multiplied by staining intensity (ranging from 0 to 3). The marker level was determined using the median H score: low if the H score is inferior to the median and high otherwise. Lobular carcinomas were excluded from CDH1 statistical analyses, as they show constitutive loss of CDH1 expression. Stromal and epithelial compartments were assessed using morphological criteria defined by a clinician. We also used HES and CDH1 staining to better distinguish epithelial and stromal cells.

Tertiary lymphoid structures per section of tumor were counted on H&E sections. For quantification of immune infiltrate, at least three fields of 1.65 mm^2^ per tumor were evaluated (× 10 magnification). We counted the number of stained immune cells of interest in each compartment (considering the epithelial and stromal compartments separately) and divided by the total area of the observed section. Both stained cells quantification and tumor infiltrating lymphocytes (TILs) on HES stained slides were evaluated according to the International TILs Working Group [35].

For quantification of vascularization, we analyzed eight fields of 1596 mm^2^ per tumor (× 10 magnification). We used ImageJ software in order to evaluate the percentage of the surface of the field stained with the CD31 antibody. Each percentage of CD31^+^ area was normalized on the percentage of stroma in the same photograph. Finally, we expressed results as the mean of the percentage of CD31^+^ area normalized by the stromal proportion.

### Decision tree algorithm for prediction of CAF subset identity

CAF identity of each tumor was determined using an algorithm developed by the team [22, 23]. In brief, the algorithm takes as input histological scores of CAF markers. Initially, the thresholds (quartiles and median) and the order of decisions were established from FACS data in a prospective cohort of BC patients, and next transposed to values of IHC data using a learning set of tumors containing both non-activated and activated CAF, as described in [22]. The decision tree algorithm is depicted in Fig. 3g.

### Maps of CAF subsets at cellular level

IHC staining from consecutive sections were scanned on Philips Ultra-Fast Scanner. The × 10 images of CAF markers used in the decision tree algorithm (CD29, FAP, SMA, FSP1) were analyzed from the same areas of representative tumors (considering controls and cases). Images were aligned using elastic transformation from Fiji software plugin (bUnwarpJ). This plugin uses landmarks manually defined on hematoxylin and eosin (H&E) staining of the sections to compute the optimal correlation between original images and alignment at cellular level by elastic transformation. Images were divided into tiles of 225 μm^2^ to mimic the approximate size of one fibroblast, and each tile was annotated according to its position in the section. Aligned and annotated images of the CAF markers were then submitted to color deconvolution, and the intensity of each DAB staining was measured by densitometry analysis using ImageJ software. Each tile was classified into a specific CAF subset using the algorithm developed by the team (see paragraph above), which takes as input DAB intensities of CAF markers measured within each tile. Epithelial tumor cells were masked (represented in gray or black) to better visualize the stromal compartment. Each tile was colored according to classification into CAF-S1 to CAF-S4, using the scheme colors defined throughout the study: CAF-S1 red, CAF-S2 orange, CAF-S3 green, and CAF-S4 blue.

### Prosigna™ assay

We performed a Prosigna™ test (Prosigna™, NanoString Technologies, Seattle, WA, USA) for all samples. We chose the Prosigna™ genomic signature because it is the only test to provide BC molecular subtype, and its long-term prognostic value (risk of distant recurrence) has been clearly validated [14, 36, 37]. For all patients, a breast pathologist identified the invasive tumor area and assessed tumor cellularity on an H&E section. According to the manufacturer’s instructions, depending on the surface area of the tumor a series of 10 μm sections were mounted onto Superfrost slides. RNA extraction and nCounter analysis were performed according to the Prosigna*™* instructions, in IC pharmaco-genomic platform. The test failed for 5 patients due to poor RNA quality or insufficient quantity. We obtained results for 99 patients in terms of molecular subtype of BC, ROR score, risk category, and 10-year distant recurrence risk.

### RNA sequencing analysis

We used RNA sequencing from CAF subsets isolated from BC (data deposited at the European Genome-phenome Archive under accession number EGAS00001002508) [22, 23], in order to examine CDH11 RNA expression.

### Human BC cell lines and cell culture conditions

We used human luminal BC cell lines MCF7 and T47D (from ATCC). Cell line identity has been verified by short tandem repeat (STR) DNA profiling. Cells were grown in DMEM (Gibco #11995) for MCF7 and RPMI (GE SH30027.01) for T47D with glucose (4.5 g/L), 4 mM L-glutamine, 1 mM sodium pyruvate supplemented with 10% fetal bovine serum (FBS) (Biosera #FB-1003/500), 1% penicillin-streptomycin (Gibco #15140122) in a humidified atmosphere of 20% O_2_, 5% (v/v) CO_2_ in air at 37 °C. Cells were tested for absence of mycoplasma contamination.

### Isolation and culture of human primary CAF-S1 fibroblasts

Primary CAF-S1 fibroblasts were obtained as described in [38]. Briefly, fresh luminal BC from different patients were received after surgery and sorted by FACS in order to keep only the CAF-S1 subset. CAF-S1 subset was defined in FACS by CD29^Med^ FAP^Hi^ FSP1^Low-Hi^ αSMA^Hi^ PDGFRβ^Med-Hi^ [22]. After selection, CAF-S1 were plated in 96-well plates and expanded in a pericyte medium (Sciencell Research Laboratories #1201) with 10% FBS (Biosera #FB-1003/500), 1% penicillin-streptomycin (Gibco #15140122) in a humidified atmosphere of 1.5% O_2_, 5% (v/v) CO_2_ in air at 37 °C. We confirmed by RNA sequencing that, after several passages in culture, CAF-S1 primary fibroblasts isolated from BC patients kept similar molecular identities as those detected without culture [38]. Moreover, we confirmed by FACS that CAF markers at protein levels corroborated data from fresh samples [38]. Functional experiments in co-culture with cancer cells were performed in DMEM with 10% FBS at 1.5% O_2_. For BC cells and primary CAF cell lines, the absence of mycoplasma contamination had been tested and confirmed.

### Silencing experiments using small-interfering RNA (siRNA)

For small interfering RNA (siRNA) experiments, CAF-S1 primary cultures were transfected with a pool of 4 specific siRNA against CDH11 (Qiagen #1027416)—siCDH11 #1 SIO2663955 ACC GTC GGA ATT CAT TGT CAA, siCDH11 #2 SI04434647 CAA GGA CAC TGT GAC CGT CAA, siCDH11 #3 SIO2663948 CAG AGA GGA TAC ATT TAA TAA, and siCDH11 #4 SI04434654 CTG AGC TGT AAT TTC GCC TTA—or non-targeting siRNA (siCTR, Qiagen #1022076, AAT TCT CCG AAC GTG TCA CGT). Transfections were carried out at a final concentration of 20 nM using DharmaFECT 1 transfection reagent (Dharmacon, #T-2001-02) according to manufacturer’s instructions. We plated 165,000 cells per well in a six-well plate and performed the transfection 24 h later.

### Immunofluorescence

The same protocol as this one recently described by the Stress and Cancer’s lab headed by Dr. F. Mechta-Grigoriou in [38] was used. In brief, 200,000 BC cells in the presence of 200,000 CAF-S1, 24 h post-silencing, were seeded on coverslips in 6-well plates. Forty-eight hours later, cells were fixed in 4% PFA for 15 min, permeabilized with 0.1% SDS in PBS for 10 min, blocked in PBS-Tween 0.1% with 5% BSA (Euromedex, #04-100-812-C) for 30 min, and incubated with antibodies diluted in PBS-Tween 0.1% with 5% BSA overnight at 4 °C. Antibodies were CDH1/E-cadherin (1:300, Cell Signaling Technology, #3195). Cells were then incubated with Cy3-anti-rabbit secondary (1:500, JacksonImmunoResearch, #711-165-152) in parallel with Alexa Fluor TM 488 phalloidin (1:200, Invitrogen, #A12379) for 30 min at RT in PBS-Tween 0.1% with 5% BSA. After several washing steps, coverslips were mounted on slides with a drop of Vectashield mounting medium with DAPI (Vector, #H-1200). Slides were next examined on an upright Epifluorescence Microscope with Apotome (Zeiss) with a × 40 oil-immersion objective and images were acquired with identical settings using a digital camera (Photometrics CoolSNAP HQ2).

### qRT-PCR

For gene expression analysis, cells were lysed in Qiazol and RNA was isolated using miRNeasy kit (Qiagen, #217004) and Qiacube, following manufacturer’s instructions. One microgram of RNA was used for reverse-transcription using random primers (iScript cDNA Synthesis kit; Bio-Rad, #170-8891). For quantitative RT-PCR (qRT-PCR), the *Power* SYBR Green PCR Master Mix (Applied Biosystems, #4367659) and primers at 500 nM (final concentration) were used in a Chromo4 Real-Time PCR detector (Bio-Rad). Primer sequences used for CDH11 are the following: reverse 5′-GGT CTG GAA CCA GTT CTT CG-3′ and forward 5′-TCT CGA TCC AAC GTC TTG GT-3′. qRT-PCR were amplified in triplicate for each sample. Expression levels were normalized to cyclophilin B and represented as fold change compared to the control (2^(− ΔΔCt)).

### Protein extracts and western blot analysis from cell lines

For protein level analyses, CAF-S1 cells were washed with PBS and scraped with Reverse Phase Protein Arra (RPPA) lysis buffer (50 mM Tris pH 6.8, 2% SDS, 5% glycerol, 2.5 mM EDTA, 2.5 mM EGTA, 4 mM Na3VO4, 20 mM NaF, and 2 mM dithiothreitol) supplemented with 2 × Halt phosphatase inhibitor (Perbio #78420) and a complete EDTA-free protease inhibitor cocktail tablet (Roche #1836170). Samples were boiled for 10 min at 95 °C. Samples were sonicated in an ice water bath for 15 min, and then centrifuged at 14,000 RPM at 4 °C for 10 min to isolate the protein supernatant and eliminate DNA contamination.

The protein concentration was determined using the BCA Assay (Thermo Fisher #23252); the samples were normalized to a concentration of 1.3 mg/ml, and then denatured for 5 min at 95 °C. For western blot analysis, 20 μg proteins were loaded onto precast 4–12% Bis-Tris protein gels (Invitrogen) with MOPS SDS (3-(N-morpholino) propanesulfonic acid sodium dodecyl sulfate) as running buffer (ThermoFisher). After electrophoresis, the proteins were transferred to a 0.45 μm nitrocellulose transfer membrane. Blocking was performed with PBS–tween and 5% of BSA during 1 h. Membranes were then blotted overnight at 4 °C with the appropriate primary antibodies diluted in 5% of BSA: CDH11 (1:500; R&D # MAB1790) and Actin (1:10,000; Sigma #A5441). Specific binding of antibodies was detected using appropriate peroxidase-conjugated secondary antibodies (Mouse, Jackson ImmunoResearch Laboratories #115-035-003) and was visualized by enhanced chemiluminescence detection (GE Healthcare Life Science). Densitometric analyses of immunoblots were performed using ImageJ software.

### Proliferation and survival assays

We plated 100,000 CAF-S1 (transfected by siCDH11 or siCTL) with 50,000 MCF7 or 75,000 T47D per well in a six-well plate (Falcon corning #353046). As a control, cancer cells were plated alone as well. The total number of cells and their survival state were then analyzed by FACS. At 72 and 168 h post-transfection, attached cells and supernatant of culture were collected, washed in PBS+ solution (PBS, Gibco #14190; EDTA 2 mM Gibco #15575; Human Serum 1%, BioWest #S4190-100), and centrifuged. Collected cells were next stained with an antibody cocktail containing Brilliant Violet 605™ anti-human EPCAM (1:50; BioLegend, #324219) for BC cells and anti-FAP-Pacific Orange (1:200; R&D Systems, #MAB3715) for 15 min at room temperature, EPCAM and FAP staining being used to distinguish CAFs from BC cells. After a washing step, all samples were resuspended in 50 μl of PBS+ containing 1:100 carboxylate beads (Polyscience #18133) and 5 μg/ml DAPI solution (Thermo Fisher scientific #D1306). Flow cytometry analyses were performed on LSRFortessa™ analyzer (BD biosciences). As FACS did not allow the analysis of the entire suspended sample, at least 5 × 10^5^ events were recorded and precision beads were used to normalize viable BC cell (DAPI^−^ EPCAM^+^ FAP^−^) and CAF (DAPI^−^ EPCAM^−^ FAP^+^) counts. Compensations were performed using single staining on anti-mouse IgG and negative control beads (BD bioscience #552843) for each antibody. Data analysis was performed using FlowJo version 10 (LLC, USA). The clustering strategy used was as follows: (i) cells were first gated based on their size (FSC-A) and their granulosity (SSC-A) to exclude debris and the number of beads was counted; (ii) cells were then gated on EPCAM and FAP expression to distinguish cancer cells (EPCAM^+^, FAP^−^) from CAF (EPCAM^−^, FAP^+^); (iii) finally, the live/dead cells were distinguished by DAPI staining.

### Transwell assay

Eight micrometers of Transwell cell culture inserts (BD Biosciences #353182) were used for Transwell assays in 12-well plates. According to the condition, BC cells (80,000 MCF7 or 120,000 T47D) with or without 20,000 CAF-S1 fibroblasts were placed on the upper part of the Transwell device and incubated in 400 μl of DMEM supplemented with 1% FBS for MCF7 and 800 μl of RPMI supplemented with 1% FBS for T47D. The lower chamber either contained 200 μl of DMEM supplemented with 1% FBS alone (control condition) or was plated with 20,000 primary CAF-S1. The experiment was stopped after 24 h of incubation at 37 °C in 5% CO_2_ and 1.5% O_2_. Medium was removed from the upper part of the Transwell device and the membrane in the upper part was washed 3 times with cotton imbibed by PBS. Next, inserts were immersed for 30 min in 4% Violet Cristal (sigma #C0775-100G) and 20% Ethanol (analaR #20821.310) for fixation and coloration. Five representative pictures per insert were taken at × 5 magnification. Pictures were then analyzed with ImageJ software. We first performed Transwell assays using only BC cells or only CAF-S1 fibroblasts on the upper chamber. By this way, we confirmed that we could easily distinguish BC cells from CAF after their migration through the Transwell, according to their respective morphological characteristics. Thus, we considered that upon cocultures BC cells and CAF-S1 displayed different morphologies that enabled us to distinguish them, as shown in Fig. 4 for cancer cells, and Additional File 8: Fig. S6 for CAF.

### Statistical analysis

All statistical analyses and graphs were performed using R environment (https://cran.r-project.org, version R-3.6.2) and RStudio (https://rstudio.com/products/rstudio/download/, version 1.2.5033). Qualitative data (TNM stage, histological subtype, CAF subsets…) were analyzed using the Fisher’s exact test. Quantitative data (age, BMI, size of tumor, immune cell content…) were first tested for their normality using the Shapiro test and then analyzed with the pertinent test (Wilcoxon-Mann-Whitney test or Student’s *t* test). Quantitative data were expressed in the manuscript as median (minimal – maximal values). Spearman’s correlation test was used to evaluate the correlation coefficient between two parameters. Data shown in this paper are generally represented as boxplots with median, 25th and 75th for the lower and upper hinges, and 1.5 × inter-quartile range from the hinge. Data from in vitro experiments were shown using mean ± SEM (unless otherwise specified) from at least three independent experiments.

Survival curves were constructed using the Kaplan-Meyer method implemented in R package *survival*. Distant progression-free survival was defined as the time between treatment and diagnosis of distant metastasis (excluding local or regional lymph node recurrence). Survival analyses were done according to the Cox model with Log-Rank test. Explanatory variables with a *p* value < 0.20 in univariate analyses were kept for the multivariate analyses using the Cox model.

Two-sided tests with *p* value < 0.05 were considered statistically significant.

## Results

### Establishment of a case-control cohort of luminal breast cancer patients

In this study, we aimed to define the cancer cell-autonomous and non-cancer cell-autonomous mechanisms involved in distant relapse in luminal BC. To do so, we built a cohort of patients from a large group of 57,665 BC patients treated at the Institut Curie (IC) Hospital Group from 1980 to 2010. In order to conduct a case-control study, we first selected 3739 female patients with unifocal invasive BC, T1b, or T1c N0M0, expressing ER without amplification of HER2, and treated by primary surgery between 2003 and 2010 (Additional File 1: Fig. S1). Survival analyses showed that those patients displayed a recurrence rate (Additional File 1: Fig. S1) that was in accordance with the literature [39]. Among these patients, 52 cases of early luminal BC with distant relapse and 52 controls without relapse were selected with a match on age, grade, and Ki67 (Table 1), features described as main prognostic factors for these patients [9, 14]. In keeping with the paired selection of patients on Ki67 staining (Table 1), we confirmed the absence of difference in tumor cell proliferation between subgroups by using the mitotic index (Table 1). In this cohort of patients, the luminal BC subtype was first determined by ER and HER2 profiling using immunohistochemistry (IHC) (Table 1), as commonly used in clinical practice [5, 6]. The luminal subtype was next validated in most patients using Prosigna™ test except for five patients. Using Prosigna™ test, four patients (2 cases and 2 controls) were classified in the HER2 subtype and one case was identified as a “basal-like” subtype. This was in accordance with the rate of discordance between IHC and Prosigna™ molecular subtype classifications reported in the literature recently [14]. A new independent evaluation of ER, progesterone receptor (PR) and HER2 IHC staining, was thus re-performed for those five patients. Their luminal identity was confirmed, but we noticed a high-proliferation rate: median of 50 mitotic figures evaluated on 10 successive high-magnification fields (min 14, max 60) and median of 50% of stained cells by Ki67 (min 20%, max 60%). These patients were thus kept in the study as initially defined as luminal subtype by IHC, but we maintained the information in all figures to avoid any potential misinterpretation of the results.

### Distant relapse was associated with high ROR score and reduced cancer cell differentiation

**Fig. 1:**
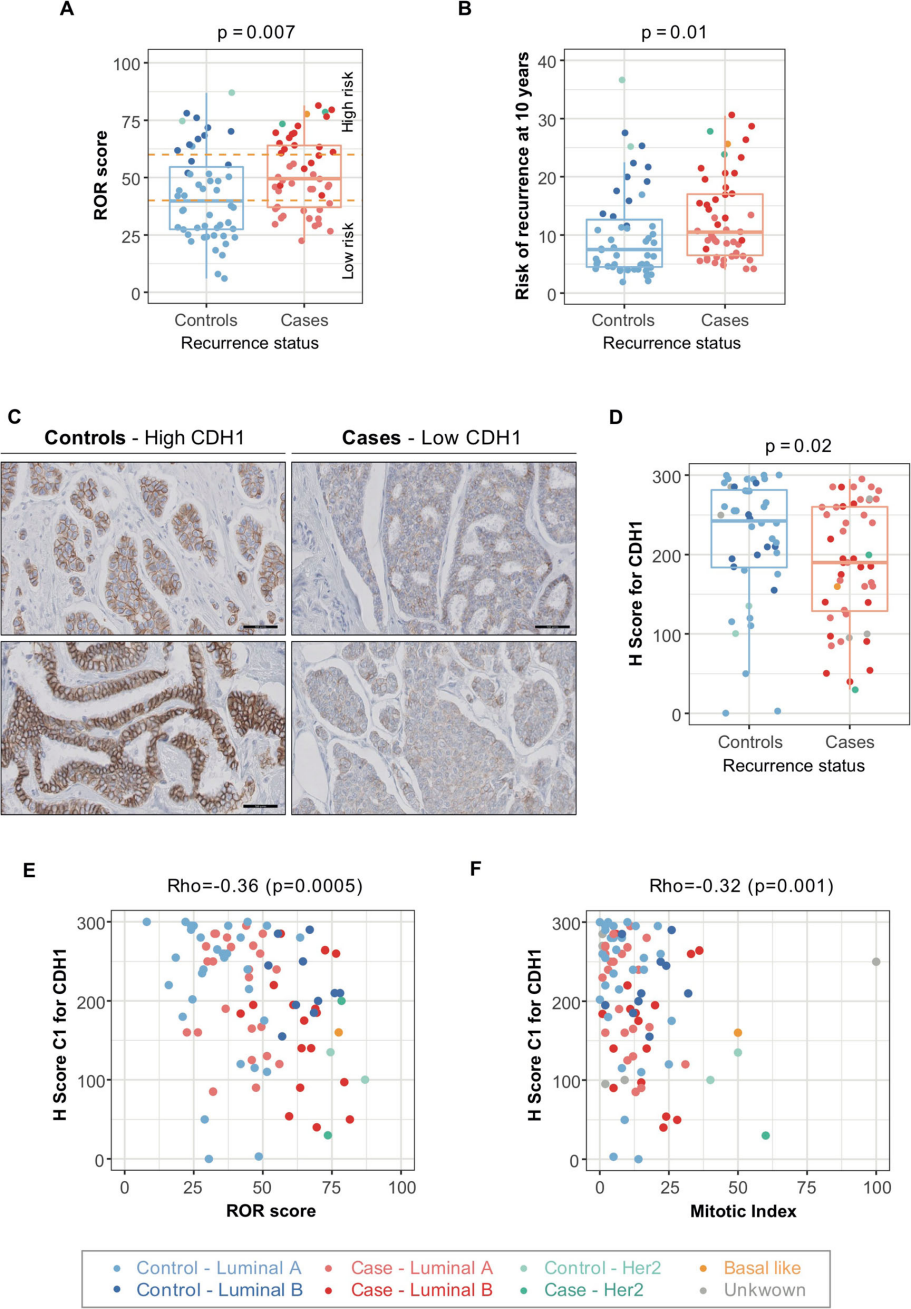
Patients with distant relapse exhibit lower CDH1 protein levels and higher Prosigna™ score than controls. **a**, **b** ROR score (**a**) and risk of distant recurrence at 10 years (**b**) assessed by Prosigna™ according to the recurrence status (*N* = 99, 49 controls, 50 cases). Horizontal dashed lines split patients according to the risk category, as defined by the Prosigna™ test as low, intermediate or high risk. *p* values are from Wilcoxon tests. **c** Representative views of CDH1 (E-cadherin) immunostaining in controls (left) and cases (right) (scale bar = 50 μm). **d** Boxplot showing CDH1 H score according to the recurrence status and the molecular subtype (*N* = 92, 44 controls, 48 cases). H score were given as a function of percentage of stained cancer cells multiplied by its respective staining intensity (ranging from 0 to 3). **e**, **f** Correlations between CDH1 H score and mitotic index (count of mitotic figures in 10 consecutive high-magnification fields) (**e**) (*N* = 92, 44 controls, 48 cases) or between CDH1 H score and ROR score (**f**) (*N* = 87, 42 controls, 45 cases). *p* values are from Spearman’s correlation tests. Lobular carcinomas (*N* = 12, 8 controls, 4 cases) were excluded from all CDH1 analyses due to their constitutive loss of CDH1 expression. The color code for boxplots and correlation plots is indicated below the figure. It depicts the BC subtype assessed by Prosigna™ test. Luminal A BC are in light blue (controls, *N* = 37) and light red (cases, *N* = 27). Luminal B BC are in dark blue (controls, *N* = 11) and dark red (cases, *N* = 19). HER2-enriched BC are in light green (controls, *N* = 2) and dark green (cases, *N* = 2). The basal-like BC is in yellow. BC without result for Prosigna™ test are in gray (2 controls and 3 cases)

We first investigated the cancer cell properties that might be linked to distant relapse in luminal BC patients. The ROR score and the risk of recurrence, defined by the Prosigna™ test, were higher for cases than controls with a median risk of distant recurrence at 10 years of 10.5% (4 to 30.5%) for cases and 7.5% (2 to 36.5%) for controls (Fig. 1a, b). Moreover, distant relapse was associated with lower CDH1 (E-cadherin) protein level assessed by histological scoring (Fig. 1c, d). This finding confirmed previous observations [40–42] but, to our knowledge, had not been heretofore shown in the T1N0 luminal BC patient subpopulation. As reduced CDH1 expression was associated with tumor size, lymph node status, and TNM stage [40–42], we subsequently investigated if CDH1 histological score (H score) was associated with any other clinical parameters. CDH1 H score was significantly anti-correlated with both mitotic index and ROR score (*p* = 0.001 and *p* = 0.0005 respectively, Spearman test) (Fig. 1e, f). Using multivariate analyses, we showed that reduced CDH1 H score was associated with an increased risk of recurrence, independent of the mitotic index (hazard ratio (HR) = 1.20; CI95% [1.01–1.43]; *p* = 0.03) (Additional File 3: Table S2). In contrast, CDH1 H score and ROR risk were not independent variables for predicting patient survival (Additional File 3: Table S2), most probably because ROR score is indicative of cancer cell proliferation as well as hormonal signaling and cell differentiation.

### Infiltration by CD4^+^ T lymphocytes is lower in cases than in controls

**Fig. 2:**
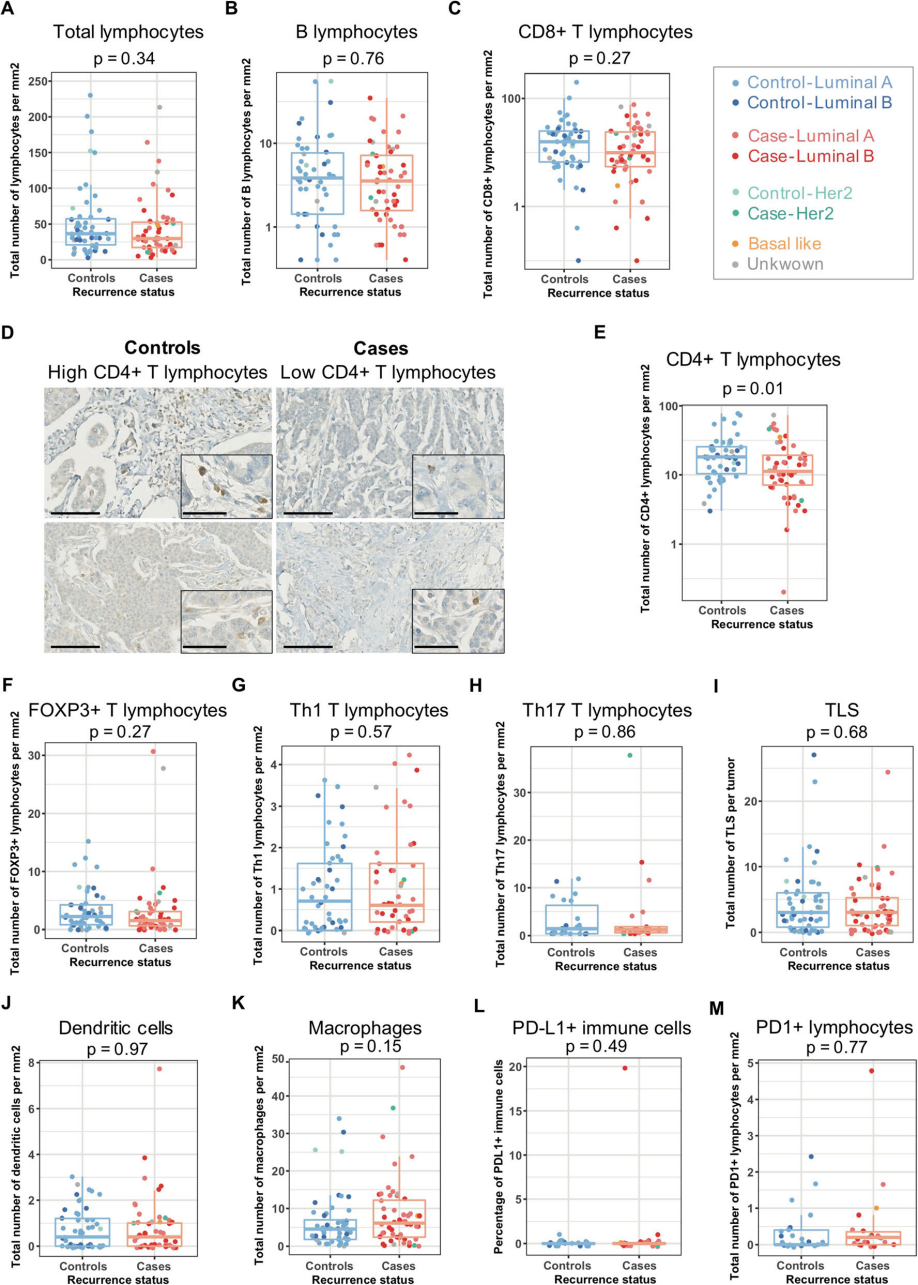
Distant recurrence in luminal breast cancer is not associated with strong modulation of immune infiltration. Boxplots showing total count of stained cells per mm^2^ for lymphocytes on HES sections (**a**), B lymphocytes using CD20 marker (**b**), CD8^+^ T lymphocytes (**c**), CD4^+^ T lymphocytes (**e**), FOXP3^+^ T lymphocytes (**f**), Th1 T lymphocytes using Tbet marker (**g**), Th17 T lymphocytes using IL17 marker (**h**), TLS count on HES sections (**i**), dendritic cells using DC Lamp marker (**j**), macrophages using CD163 marker (**k**), PD-L1^+^ immune cells (**l**), and PD-1^+^ lymphocytes (**m**) according to the recurrence status and the molecular subtype. *N* = 104 patients (52 controls and 52 cases) for all immune analyses except for PD-1 and PD-L1 markers (*N* = 44, 22 controls, 22 cases). **d** Representative views of CD4 + T lymphocytes immunostaining in either controls (left) and cases (right) (scale bar = 100 μm for larger views and 50 μm for inserts). *p* values are from Wilcoxon tests. The color code for boxplots is indicated at the top right of the figure. It depicts the BC subtype assessed by Prosigna™ test. Luminal A BC are in light blue (controls, *N* = 37) and light red (cases, *N* = 27). Luminal B BC are in dark blue (controls, *N* = 11) and dark red (cases, *N* = 19). HER2-enriched BC are in light green (controls, *N* = 2) and dark green (cases, *N* = 2). The basal-like BC is in yellow. BC without result for Prosigna™ test are represented in gray (2 controls and 3 cases)

Having identified some cancer cell features (such as CDH1 H-score and ROR score) associated with distant relapse in luminal BC, we next tested variation in TME composition that could be linked to distant relapse in these patients. We analyzed two main components of the TME (namely immune and stromal cells) and investigated their global content and diversity (including lymphocytes subtypes and CAF subsets). Analyses were performed within the tumor bed regarding the immune infiltration at the surface of either epithelial or stromal cells. We observed that the total number of tumor-infiltrating lymphocytes and tertiary lymphoid structures (TLS) were similar between cases and controls (Fig. 2a, b). Interestingly, there was a lower CD4^+^ T lymphocyte infiltrate in cases compared to controls: median of 11.2 CD4^+^ T lymphocytes per mm^2^ (0.2 to 73.3) for cases and 18.2 CD4^+^ T lymphocytes per mm^2^ (3.0 to 77.4) for controls (Fig. 2d, e). CD4^+^ T lymphocyte content was correlated with the CDH1 H score in cases, while this was not the case in controls (Rho = 0.21, *p* = 0.044 in the whole cohort; Rho = 0.36, *p* = 0.009 for cases, Rho = − 0.03, *p* = 0.83 for controls, Spearman test), suggesting that, for cases, tumor cell differentiation state is linked with CD4+ T cell infiltration. In contrast, CD4^+^ T lymphocyte content was not correlated with ROR score (Rho = − 0.16, *p* = 0.09, Spearman test). Contrary to CD4^+^ T lymphocytes, the content in all other immune cells, including cytotoxic (CD8^+^), regulatory (FOXP3^+^), Th1 (T-Bet^+^), Th17 (IL-17^+^) T lymphocytes, B lymphocytes (CD20^+^), mature dendritic cells (DC-LAMP^+^), macrophages (CD163^+^), PD-L1^+^, and PD-1^+^ lymphocytes, was equivalent between cases and controls (Fig. 2c, f–m). Moreover, no PD-L1 staining was detected in epithelial cells, neither in controls nor in cases, thereby precluding any impact of PD-L1 expression in tumor cells in luminal BC at diagnosis for ulterior relapses. As recently reported [22, 23], we observed that the total account of lymphocytes was much lower at the surface of epithelial cancer cells compared to stromal fibroblasts. Indeed, 0 to 12.8% of the total lymphocyte account was detected at the surface of the epithelium compared to the stroma. Although there was no difference between cases and controls in the content of intra-epithelial immune cells (regardless of population studied) (Additional File 4: Fig. S2), the proportion of CD4^+^ T lymphocytes at the surface of the stroma was significantly lower in cases than in controls (Additional File 4: Fig. S2) (*p* = 0.01, Wilcoxon test). Based on that observation, we next wondered if the difference in CD4^+^ T lymphocyte infiltration between cases and controls could be due to a distinct vascularization pattern. Using CD31 immunostaining of vascular endothelial cells, we observed higher vascularization in cases than in controls with a median stromal surface of vessels of 1.96% (0.22–11.38%) for cases and 1.27% (0.30–14.24%) for controls (*p* = 0.009, Mann-Whitney test, Additional File 5: Fig. S3). Hence, we could conclude that the lower rate of CD4^+^ lymphocyte infiltration in cases was not due to reduced vascularization.

### Cases are enriched in the activated CAF-S1 subset

**Fig. 3:**
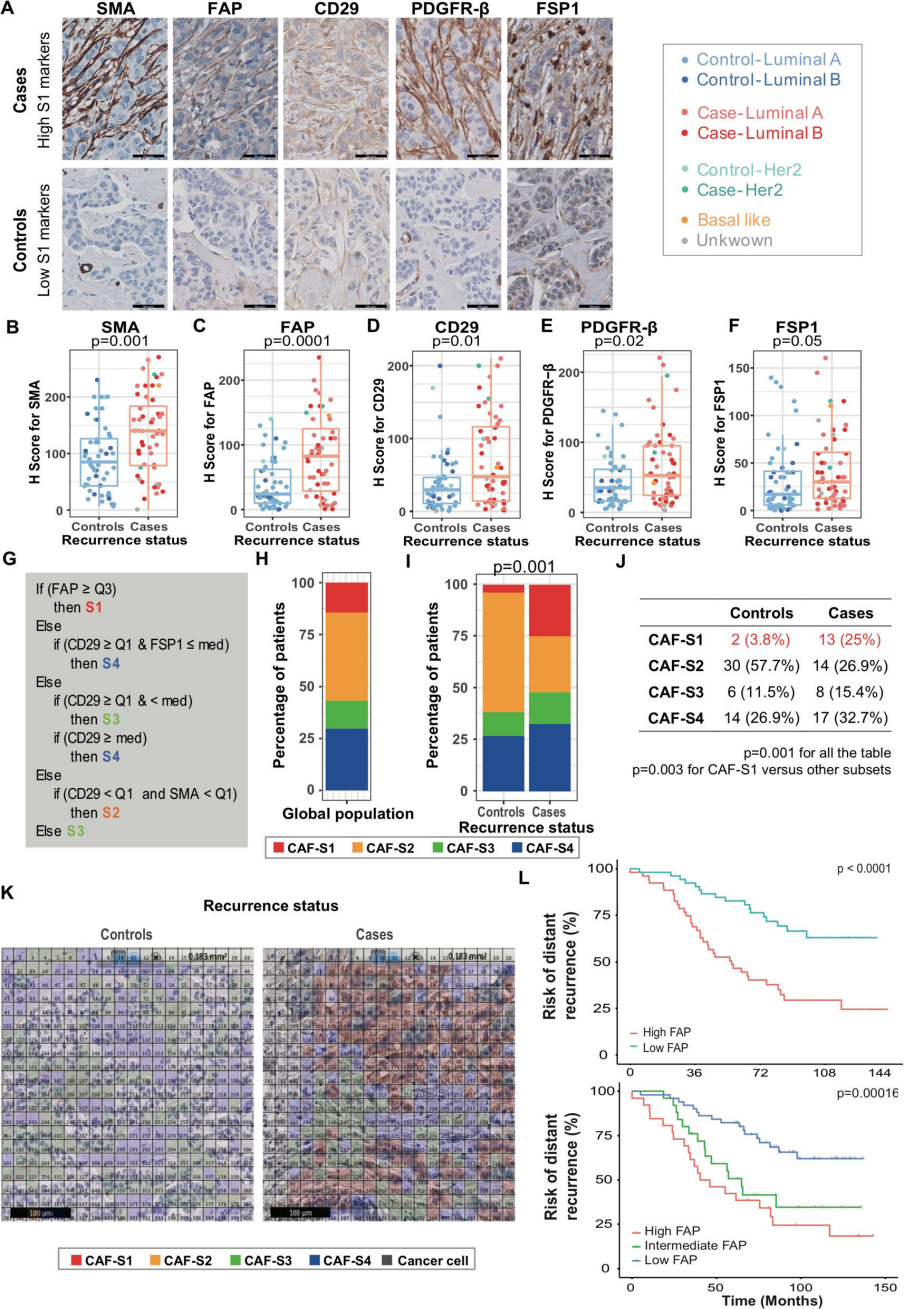
A specific subset of CAF is associated with distant recurrence in luminal breast cancer. **a** Representative views of SMA, FAP, CD29, PDGFR-β, and FSP1 immunostaining in cases and controls (scale bar = 50 μm). **b**–**f** Boxplots showing histological scores (H scores) for CAF markers (SMA, FAP, CD29, PDGFR-β, and FSP1) according to recurrence status and molecular subtype (*N* = 104, 52 controls, 52 cases). H scores are given as a function of percentage of stained CAF multiplied by its respective staining intensity (ranging from 0 to 3). *p* values are from Wilcoxon tests. Boxplots color code is indicated below **b**–**f**. It indicates the BC subtype d by Prosigna™ test. Luminal A BC are in light blue (controls, *N* = 37) and light red (cases, *N* = 27). Luminal B BC are in dark blue (controls, *N* = 11) and dark red (cases, *N* = 19). HER2-enriched BC are in light green (controls, *N* = 2) and dark green (cases, *N* = 2). Basal-like BC are in yellow. BC without result by Prosigna™ test are in gray (2 controls and 3 cases). **g** Decision tree used to define CAF subset identity according to CAF maker intensities, based on 4 equal quartiles (Q1-Q4) and median (med) distribution of each CAF marker, as shown in [22]. **h**, **i** Bar plot showing the distribution of CAF subset enrichment in luminal early BC for all patients (**h**) and according to the recurrence status (**i**). For each BC analyzed, enrichment is defined by applying H scores of all markers on the decision tree described in **g**. Breast cancer enriched in CAF-S1 (red), CAF-S2 (orange), CAF-S3 (green), or CAF-S4 (blue) are shown as percentage (%) (*N* = 104, 52 controls, 52 cases). *p* value is from Fisher exact test. **j** Contingency table for the repartition of CAF subsets enrichment according to the recurrence status (*N* = 104, 52 controls, 52 cases). *p* values are from Fisher exact test. **k** Representative views of maps of CAF subsets at cellular scale using the decision tree algorithm (shown in **g**) and CAF marker histological scoring on serial BC sections (corresponding sections and staining shown Additional File 6: Fig. S4). CAF-S1 are in red, CAF-S2 in orange, CAF-S3 in green, and CAF-S4 in blue. Scale bar, 100 μm. **l** Left, Kaplan-Meier survival analysis for distant recurrence rate according to low- and high-FAP H scores. Patient subgroups defined by median. *p* value is from Log-Rank test. Right, same as in left with patients stratified in 3 categories according to FAP H scores: low-FAP if H score ≤ 47 (*N* = 52), intermediate-FAP if H score > 47 and ≤ 95 (*N* = 26), and high-FAP if H score > 95 (*N* = 26). *p* value is from Log-Rank test

As there was no clear difference in immune infiltration between cases and controls (except for CD4^+^ T cells), we next aimed to investigate the potential link between fibroblastic stroma and distant relapse in luminal BC. Indeed, while fibroblasts remain one of the most abundant components of the TME, the impact of stromal quantity and heterogeneity on relapse is far from known. We thus performed a global analysis of the stroma, considering both its quantity and its quality, in particular by assessing the repartition of the 4 CAF subsets recently identified in human breast and ovarian cancer [22, 23]. Although the median percentage of intratumoral stroma was 50%, the stromal content was not significantly different between controls and cases (*p* = 0.73, Mann-Whitney test, Additional File 6: Fig. S4). Similarly, using a semi-quantitative score, we found that the proportion of adipose stroma was equivalent between controls and cases (*p* = 0.78, Additional File 6: Fig. S4). We thus evaluated the distinct CAF subsets recently identified [22, 23]. To do so, we analyzed concomitantly the protein level of the same stromal markers as those described in previous studies [22, 23], enabling us to identify the 4 CAF subpopulations. These stromal markers were FAP (fibroblast activation protein α1), αSMA, integrin β1/CD29, S100-A4/FSP1 (fibroblast-specific protein 1), and PDGFRβ (platelet-derived growth factor receptor-β). CAF subsets have been previously defined as follows: CAF-S1: CD29^Med^ FAP^Hi^ FSP1^Low-Hi^ αSMA^Hi^ PDGFRβ^Med-Hi^; CAF-S2: CD29^Low^ FAP^Neg^ FSP1^Neg-Low^ αSMA^Neg^ PDGFRβ^Neg^; CAF-S3: CD29^Med^ FAP^Neg^ FSP1^Med-Hi^ αSMA^Neg-Low^ PDGFRβ^Med^; CAF-S4: CD29^Hi^ FAP^Neg-Low^ FSP1^Low-Med^ αSMA^Hi^ PDGFRβ^Low-Med^ [22, 23]. To highlight the content in the different CAF subsets, we performed IHC using the 5 aforementioned stromal markers on serial sections from controls and cases (Fig. 3a). All CAF-S1 and CAF-S4 markers accumulated more in cases than in controls: αSMA, *p* = 0.001; FAP, *p* = 0.0001; CD29, *p* = 0.01; PDGFRβ, *p* = 0.02; FSP1, *p* = 0.05 (Mann-Whitney tests) (Fig. 3b–f). Using a decision tree based on the histological staining of CAF markers developed in [22, 23] and described in (Fig. 3g), we found that early luminal BC were mostly enriched in CAF-S2 subset (42.3%), followed by CAF-S4 (29.8%), CAF-S1 (14.4%), and CAF-S3 (13.5%) (Fig. 3h). Interestingly, cases were significantly enriched in the CAF-S1 subset that reached 25% for cases compared to 3.8% for controls (*p* = 0.003, Fisher test comparing CAF-S1 versus other subsets, in cases versus controls) (Fig. 3i, j). Finally, we sought to visualize CAF subsets at a cellular level in both controls and cases. As we could not perform single cell analysis using fresh samples in this rare T1N0 luminal BC patient cohort, we developed an image analysis tool at cellular level that combined spatial registration and computational analysis on serial consecutive IHC sections (shown in Additional File 6: Fig. S4; see Methods, #*Maps of CAF subsets at cellular level*). We applied the CAF decision tree algorithm (described in Fig. 3g) determining CAF subset identity and generated maps of CAF subsets at cellular level (Fig. 3k). In this manner, we could visualize CAF subset spatial distribution in T1N0 luminal BC. Representative pictures of CAF subset maps confirmed the overall enrichment in CAF-S1 in cases compared to controls (Fig. 3k).

Importantly, using multivariate analyses by the Cox proportional hazard model, we observed that distant recurrence in early luminal BC was associated with the CAF-S1 subset (HR = 5.02; CI95% [2.34–10.73]; *p* < 0.0001), independent of any other features analyzed, such as CDH1 H score, ROR risk category, CD4^+^ T lymphocyte infiltration, and macrophage infiltration and vascularization (Table 2). Among the distinct parameters analyzed, cancer cell characteristics, including CDH1 protein levels and ROR risk, either remained significant but with low pertinence (CDH1: HR 1.23; CI95% [1.007–1.51] *p* = 0.042) or even lost significance (ROR risk) in multivariate analyses (Table 2), indicating that they are not the most significant indicators of relapse in luminal BC patients. In contrast, among TME features, the CAF-S1 subset remained the most significant one in multivariate analyses (Table 2), indicating that the CAF-S1 subset is a crucial component in relapse of luminal BC. We confirmed the impact of the CAF-S1 content on distant recurrence by using FAP histological scoring, as a specific marker of CAF-S1 quantity per tumor. Interestingly, patients with a high level of FAP showed an earlier distant recurrence rate than patients with a low level of FAP (Fig. 3l). We obtained similar results when we divided BC patients into three subgroups, with a better prognosis for low-FAP H score and faster distant recurrence for intermediate- and high-FAP subgroups of patients (Fig. 3l). Taken as a whole, these data demonstrate a “dose-effect” relationship between CAF-S1 content and distant recurrence risk in luminal BC patients.

**Table 2:** Univariate and multivariate analyses for using Cox model for risk of distant relapse according to cancer cell and tumor microenvironment features

	Univariate analyses			Multivariate analyses		
	HR	CI95% []	*p* value	HR	CI95% []	*p* value
**CDH1**						
Range 250–300	Ref	–	–	Ref	–	–
Per decrease of 50 pts	1.21	[1.03–1.44]	0.02	1.23	[1.007–1.51]	0.04
**ROR risk category**						
Low risk	Ref	–	–	Ref	–	–
Intermediary risk	1.98	[0.98–4.00]	0.05	1.68	[0.75–3.76]	0.20
High risk	2.45	[1.20–4.98]	0.01	1.62	[0.67–3.91]	0.28
**CAF subset**						
S1	3.98	[2.09–7.59]	< 0.0001	5.02	[2.34–10.73]	< 0.0001
S2, S3, or S4	Ref	–	–	Ref	–	–
**CD4**^**+**^**lymphocytes**						
Low	1.88	[1.07–3.30]	0.02	2.973	[1.50–5.88]	0.001
High	Ref	–	–	Ref	–	–
**Macrophages**						
Low	Ref	–	–	Ref	–	–
High	1.84	[1.04–3.22]	0.03	1.60	[0.83–3.09]	0.15
**CD31**^**+**^**vessels**						
Low	Ref	–	–	Ref	–	–
High	1.73	[0.99–3.07]	0.05	2.56	[1.32–4.97]	0.005

Level of markers was determined using the median H score

Lobular carcinomas were excluded from analysis using E-cadherin (*N* = 12)

*CAF* cancer-associated fibroblasts, *CI* confidence interval, *HR* hazard ratio, *Pts* points

### Cadherin 11 expression by CAF-S1 enhances cancer cell pro-tumorigenic properties

**Fig. 4:**
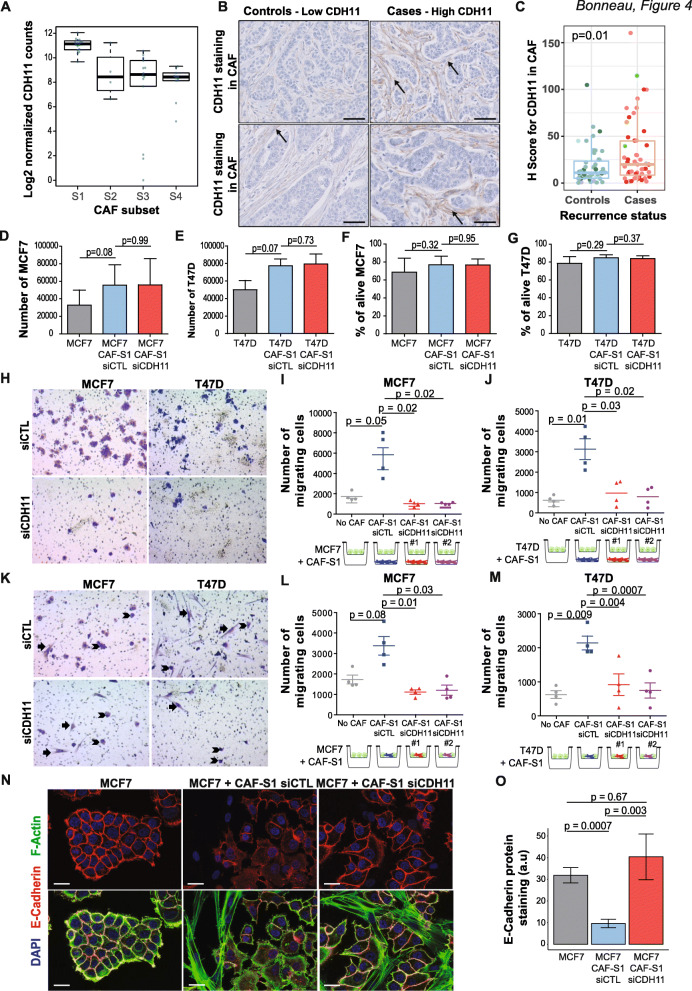
CDH11 expression by CAF-S1 enhances cancer cell migratory properties. **a** CDH11 mRNA levels in CAF subsets (CAF-S1 to S4) from BC of Curie cohort described in [22]. Data expressed in log2^+1^ are from RNAseq and shown as mean ± SEM. **b** Representative views of CDH11 immunostaining in stromal compartment in controls (left) and cases (right) (scale bar = 50 μm). **c** CDH11 H score in CAF according to recurrence status (*N* = 102, 51 controls, 51 cases). *p* value is from Wilcoxon test. **d**, **e** Number of MCF7 (**d**) and T47D (**e**) cancer cells alone or after 3 days of co-culture with CAF-S1 transfected with non-targeting siRNA (CAF-S1 + siCTL) or with CDH11-targeting siRNA (*N* = 3, each in triplicates), after normalization on carboxylate beads. **f**, **g** Same as in **d**, **e** for percentage of alive MCF7 (**f**) and T47D (**g**) cancer cells assessed by FACS analysis (DAPI^−^) (*N* = 3 in triplicates). **h**–**j** Migration of MCF7 (**d**) and T47D (**e**) cancer cells (evaluated by Transwell assays) without (**h**–**j**) or with (**k**–**m**) contact with CAF-S1 transfected with control or CDH11-targeting siRNA. **h** Representative images of lower-side Transwell using MCF7 (left) and T47D (right) into the well and CAF-S1 transfected with control or CDH11-targeting siRNA. **i**, **j** Number of cancer cells (MCF7 (**i**) and T47D (**j**)) that have migrated in Transwell lower-side. Data are means ± SEM (*N* = 4). Schemas show Transwell designs used, with two different siCDH11 tested (siCDH11#1 and siCDH11#2). (K-M) Same as in **h**–**j** with CAF-S1 cultured in contact of cancer cells. In **k**, arrowheads indicate cancer cells and arrows CAF-S1, distinguished by morphological criteria. (*N* = 4). **n** Images of E-cadherin (red) and DAPI (blue) staining (top) or of E-cadherin (red), F-actin (green), and DAPI (blue) co-staining (bottom) in BC cells alone or in presence of CAF-S1 transfected with control or CDH11-targeting siRNA. Scale bars, 20 μm. **o** Quantification of E-cadherin staining per BC cell area (at least six images analyzed per condition). Data are mean ± SEM) (*n* = 3). a. u., arbitrary units. *p* values from Mann-Whitney test

As the CAF-S1 subset is one of the main indicators of relapse in luminal BC, we set about identifying the molecular mechanism involved. We first used RNA sequencing analysis from CAF subsets isolated from BC (data deposited at the European Genome-phenome Archive under accession number EGAS00001002508) [22] and found that CDH11 (cadherin 11, also referred to as osteoblast cadherin) was significantly up-regulated in CAF-S1 subset compared to the other CAF subsets (Fig. 4a). CDH11 is involved in bone development and is expressed in cells with a mesenchymal phenotype [43–47]. Luminal BC mainly relapse in the bone [48, 49]. This was confirmed in our cohort with 41 cases (78.8%) showing bone metastases during their evolution. We thus hypothesized that high CDH11 expression in CAF-S1 cells could explain, at least in part, the distant relapse in luminal BC. We performed CDH11 IHC on sections of our cohort and observed that CDH11 was mostly detected in CAF and significantly more expressed in the stromal compartment in cases than controls (*p* = 0.01) (Fig. 4b, c). Moreover, CDH11 was also significantly higher in tumors enriched in CAF-S1 compared to others (*p* = 3.73e−06) (Additional File 7: Fig. S5). In contrast, CDH11 expression was weak or even undetectable in tumor cells and without any difference between cases and controls (Additional File 7: Fig. S5). In addition, while 29% of patients with low CDH11 H score in stroma showed bone metastases, this proportion reached 49% in patients with high-CDH11 stromal H score (*p* = 0.06 by Fisher exact test, Additional File 7: Fig. S5). Although this result did not reach significance at 5%, it showed enrichment close to significance. This led us to assume that high CDH11 expression in CAF-S1 could be involved, at least in part, in bone relapse in T1N0 luminal BC patients. We thus performed in vitro functional assays to decipher the crosstalk between luminal cancer cells and CAF-S1 fibroblasts and investigate the impact of CDH11 silencing into CAF-S1 cells (see Additional File 8: Fig. S6 for silencing efficiency) on cancer cell features. We first verified that CDH11 silencing had no effect on CAF-S1 proliferation rate and survival (Additional File 8: Fig. S6) and next studied its impact on two BC cell lines, i.e., MCF7 and T47D, well-known for their luminal properties. Moreover, we verified that CAF-S1 primary fibroblasts isolated from BC patients exhibited the same molecular identity as CAF-S1 fibroblasts without culture, as demonstrated in [38]. After 3 days of co-culture, CAF-S1 primary fibroblasts tended to increase MCF7 and T47D proliferation, although without reaching significance; and CDH11 silencing in CAF-S1 had no impact on the total number of cancer cells (Fig. 4d, e). Similarly, the survival rate of these two cancer cell lines was not affected by CDH11 silencing in CAF-S1 cells (Fig. 4f, g). Importantly, using Transwell assays we observed that, in close contact or even at distance, CAF-S1 fibroblasts increased the migration of MCF7 and T47D luminal BC cell lines (Fig. 4h–m). Interestingly, CDH11 silencing in CAF-S1 significantly inhibited the migration of these luminal BC cells (Fig. 4h–m), highlighting the impact of CDH11 in CAF-S1 cells on the migratory capacity of luminal BC cells. We could not exclude that, upon co-culture with CAF-S1 cells (as in Fig. 4h–j), BC cells initiated a first step of epithelial-to-mesenchymal transition conferring a “mesenchymal-shape” to BC cells. However, data shown in (Fig. 4k–m) were based on the distant effect of CAF-S1 fibroblasts. In these conditions, only cancer cells were on the top part of the Transwell and strictly analyzed in terms of migration. In these experimental settings, CAF-S1 fibroblasts enhanced BC cell migration without contact, an effect which was lost when CH11 was inhibited (Fig. 4l, m). Finally, in agreement with reduced tumor cell migration upon CDH11 silencing in CAF-S1 fibroblasts, we found that these conditions had an impact on CDH1/E-cadherin protein levels in both MCF7 and T47D tumor cells. Indeed, upon co-culture of CAF-S1 and tumor cells, CAF-S1 fibroblasts significantly decreased CDH1/E-cadherin protein levels in tumor cells, as we recently demonstrated in [38]. Interestingly, we observed that CDH11 silencing in CAF-S1 prevented this effect and kept the CDH1/E-cadherin protein level elevated at the surface of MCF7 and T47D tumor cells (Fig. 4n, o and Additional File 8: Fig. S6). Maintenance of CDH1/E-cadherin protein levels in tumor cells upon co-culture with CAF-S1 could explain the reduction of tumor cell migration we observed upon CDH11 silencing in CAF-S1. These new data highlight the mechanism by which CDH11 act on cancer cell migration and give new insights into CAF-S1-mediated CDH11 function on cancer cell metastatic spread in luminal breast cancer. Thus, high expression of CDH11 in CAF-S1 fibroblasts in luminal BC increases the pro-migratory capacity of luminal BC cells, thereby giving insights of the role of CAF-S1 in distant relapse in luminal BC.

## Discussion

Although T1N0 luminal BC patients often exhibit a good prognosis, 5 to 10% of patients will relapse and ultimately die. The generally high rate of their survival explains in part the paucity of studies on these patients until now [9, 10]. Here, we address the question of distant metastases in these early luminal BC patients by combining analyses on both epithelial and stromal compartments. We found that relapse in early luminal BC is associated with both tumor cell-autonomous and non-autonomous effects. In particular, we demonstrate that one main mechanism of relapse in these patients is mediated by a specific CAF subset, referred to as CAF-S1. This CAF-S1-dependent effect on relapse is independent of the tumor cell-autonomous effects, including differentiation state and proliferation. Moreover, the CAF-S1-mediated effect on relapse provides an additive value to immune infiltrate and blood vessel vascularization. Finally, we established that the CDH11/osteoblast cadherin, which is highly expressed by CAF-S1 and accumulates in cases compared to controls, increases migration properties of luminal BC cells.

The prognosis of luminal BC, T1b-cN0, is very favorable, as confirmed in our cohort. Among the 3739 patients suitable for inclusion in the Curie cohort, only 71 patients had distant metastatic recurrence (97.8% of distant recurrence-free survival at 5 years). The “case-control” design of our cohort was specially adapted to this situation, as the occurrence of the event (i.e., metastatic recurrence) is rare. To our knowledge, this is the first “case-control” study to address the issue of metastatic recurrence in patients with small BC and generally of good prognosis. Although the number of patients with metastatic relapse is low, it is important to better understand the mechanisms and risk factors involved in recurrence. On the one hand, identification of high-risk patients might justify an increase in adjuvant treatments in order to prevent recurrence; on the other hand, this could lead to a decrease in adjuvant therapies in low risk patients to avoid treatment side effects. Importantly, none of the clinicopathological criteria commonly used in clinics (i.e., histological subtype, percentage of ER or PR, grade, size, neoplastic emboli) were significantly different between cases and controls. This may explain why patient prognosis is currently difficult to assess in clinics and reinforces the clinical value of our study.

We firstly established that reduced CDH1/E-cadherin protein level was associated with metastatic recurrence. Previous studies have already reported an association between low CDH1 expression and increased risk of recurrence [40–42, 50–56]. However, other clinical studies did not confirm this association and contradictory reports were published on the utility of CDH1 assessment as an independent prognostic marker in invasive BC [57–60]. Moreover, these studies were neither dedicated to small luminal BC nor performed on sub-stratified patients, thereby highlighting the interest of our study. The association between reduced CDH1 protein levels and recurrence risk is possibly due to the epithelial-to-mesenchymal transition [61], although some studies have shown that CDH1-mediated cell-cell adhesion can promote chemoresistance and cell survival in the blood circulation [62–65]. Still, a CDH1-mediated effect remains at the limit of significance in multivariate analysis in our study. Similarly, although the ROR score estimated by Prosigna™ test was significantly different between controls and cases, this difference was small in absolute value with poor discrimination. As the PAM50 signature used in the Prosigna™ test relies on proliferation genes, the difference between the two subgroups could be minimized by the initial adjustment on proliferation rate we used to match controls to cases [12]. This suggests that other prognostic factors identified here, such as TME components, might be more relevant than tumor cell features in luminal BC patient relapse.

Luminal BC are known to be poorly immunogenic compared to HER2 or triple-negative BC. By performing a global analysis of a large panel of cells from innate and acquired immunity and biomarkers for immunotherapies, we confirmed that T lymphocyte infiltration is rather weak (about 30 lymphocytes/mm^2^ on average) in early luminal BC. Consistent with the existing literature on the lack of prognostic value of T lymphocytes in luminal BC [66–69], we also found that the overall density in T cells or in CD8^+^ T lymphocytes was not associated with metastatic recurrence. However, we observed a lower rate of CD4^+^ T lymphocytes in patients with metastatic recurrence than in controls, an effect that needs to be confirmed in other cohorts as little evidence exists regarding the prognostic value of CD4^+^ T lymphocytes in luminal BC. Besides, PD-1 and PD-L1 expression was weak with very little inter-tumor variation. Although we could not exclude that the low PD-L1 staining in TILs was not linked to technical difficulties in maintaining good immunoreactivity of embedded tissues once sectioned [70], our findings confirmed previous data on luminal BC patients [71, 72] that showed low PD-1 and PD-L1 expression in luminal BC and were not in favor of anti-PD-1 or PD-L1 use in luminal BC type.

In contrast to the immunological content, enrichment in CAF-S1 fibroblasts is a new risk factor for recurrence in early luminal BC, independent of epithelial, immune, and vascular features. Several fibroblast markers (αSMA, FAP, CD29, PDGFRβ) are associated with metastatic recurrence. Previous studies have shown that increased expression of αSMA in BC stroma is associated with high-grade, nodal invasion, increased neovascularization, and poor prognosis [17, 73–75]. To our knowledge, the characterization of the CAF-S1 subset and its implication in distant relapse have never been established. Transcriptomic signatures of genes involved in ECM remodeling, immunity, and angiogenesis have been developed recently [22, 25, 76–82]. Most of them have been developed from cohorts including all BC types and stages. Tumor stromal prognostic value was independent of ER or HER2 expression, grade, age, chemo- or endocrine-therapy [80], highlighting the importance of stromal biology in tumor progression and patient clinical outcome. In that context, our study confirms the existence of 4 CAF subpopulations in luminal BC, as observed previously in luminal A BC [22]. Unfortunately, due to the rare occurrence of distant recurrence in the T1N0 luminal BC subpopulation, we could not confirm this finding using prospective single cell analysis from fresh tumor samples. Still, we found a particularly strong prognostic value of CAF-S1 independent of epithelial, immune, and vascular features. Moreover, we observed that CAF-S1-mediated effects were not linked to their previously described immunosuppressive function [21–24]. Here, we noted higher vascularization in cases than in controls. This increased vascularization is independent of CAF enrichment and CD4^+^ T cell infiltration, as shown by multivariate analysis. These observations are in accordance with previous data showing that high micro-vessel density was significantly associated with poor prognosis in high-grade luminal A BC patients [83]. In the NSABP B-40 clinical trial evaluating bevacizumab (anti-VEGF targeted therapy) efficiency in advanced BC, the addition of neoadjuvant plus adjuvant bevacizumab lead to a significant increase in overall survival, especially in hormone-receptor-positive BC patients [84]. Treatment exhibited a strong effect on distant metastases and its efficiency was even stronger beyond 2 years of follow-up, suggesting a predominant effect on undetectable micro-metastases present at the time of diagnosis. These data might encourage further investigation into vascular supply in luminal BC. Taken as a whole, our study is original as it deciphers the role of the TME in the rare medical situation where a patient with a very good prognosis shows, in contrast, late relapse with metastases. Hence, the novelty of our manuscript is based on the discovery of an excess risk of distant recurrence linked to the TME and not only linked to tumor cells, as it is usually described. The notion described here thus highlights that early luminal BC may relapse not only because of aggressive tumor cells but also (and more significantly) because of a permissive microenvironment.

Our study found an increased expression of CDH11 in the stroma of BC cases compared to controls, consistent with CAF-S1 accumulation. In contrast, epithelial expression of CDH11 was not different between cases and controls, while CDH11 was shown to be overexpressed in basal-like breast cancer [45–47, 85]. Using functional assays, we found that inhibition of CDH11 expression in CAF-S1 fibroblasts significantly reduced CDH1/E-cadherin expression in tumor cells and CAF-S1-mediated pro-migratory effects on BC cell lines, thereby highlighting that CDH11 is a new key player in relapse in early luminal BC. This effect was detected both in contact, as well as at a distance, suggesting the role of secreted factors by CAF-S1 in a CDH11-dependent manner. Some studies have previously highlighted the existence of a cleaved secreted CDH11 isoform [86, 87]. Although we could not detect this isoform in CAF-S1 cellular extracts and corresponding supernatants, we could hypothesize that CDH11 secreted isoform might be involved in the distant effect of CDH11 on cancer cell migration. CDH11 is already a therapeutic target in rheumatoid arthritis, an inflammatory disease with properties shared with cancer [88, 89]. Systemic administration of anti-CDH11 antibodies restricts the proliferation and migration of synoviocytes to inflammatory joints and decreases the symptoms of rheumatoid arthritis [88]. In BC, the administration of anti-CDH11 antibodies significantly inhibited the growth of the triple-negative MDA-MB-231 xenografts [47]. CDH11 inhibitors could thus be interesting target drugs for BC overexpressing CDH11 with high risk of relapse. Our work also demonstrates the urgency of having in clinical practice tools that would integrate all clinical and biological data, including tumor, immune, and fibroblastic features, to help with prognostic stratification and thus therapeutic decision-making.

## Conclusions

Our study provides one of the first deep analyses of features of tumor cells and their micro-environment associated with distant recurrence in small node-negative (T1N0) luminal BC, patients who have typically been less studied than other BC subtypes, despite their high frequency at time of diagnosis. In particular, we show that a specific subset of CAF (referred to as CAF-S1) significantly accumulates in tumors with distant relapse. Moreover, we identify CDH11 as a key player in CAF-S1-mediated pro-metastatic activity. These findings could help clinicians identify luminal BC with high risk of relapse. Moreover, targeted therapies against CAF-S1 using anti-FAP antibody or compounds targeting CDH11 might be of interest in the prevention of relapse in early luminal BC patients with stroma activation.

## Supplementary information

**Additional file 1: Fig. S1.** Related to Methods (# Patient cohort) and Table 1. Flow chart for selecting the population of BC patients studied and corresponding survival curves. Flow chart for selecting cases and controls in our cohort (A) and survival analysis of the population of interest (*N* = 3739) for the recurrence rate (B) and the distant recurrence rate (C). Briefly, the population of interest was female patients with unifocal invasive breast cancer, T1b or T1c N0 M0 (BC smaller or equal to 2 cm, without invaded lymph node and distant metastasis at diagnosis), expressing ER without overexpression of HER2, treated at Institut Curie by at least primary surgery between 2003 and 2010 (N = 3739). Survival curves were constructed according to Kaplan-Meyer method.

**Additional file 2: Table S1.** Related to Methods (# Immunohistochemistry). List of primary antibodies and immunohistochemistry conditions used in the study.

**Additional file 3: Table S2.** Related to Fig. 1. Multivariate analysis using Cox model for risk of distant relapse according to CDH1 (E-Cadherin) expression and proliferation rates.

**Additional file 4: Fig. S2.** Related to Fig. 2. Immune profiling in control luminal BC and cases with recurrence. Boxplots showing the count of stained cells per mm^2^ at the surface of epithelial (left) and stromal (right) compartments for total lymphocytes on HES sections (A), B lymphocytes using CD20 marker (B), CD8^+^ T lymphocytes (C), CD4^+^ T lymphocytes (D), FOXP3^+^ T lymphocytes (E), Th1 T lymphocytes using Tbet marker (F), Th17 T lymphocytes using IL17 marker (G), dendritic cells using DC Lamp marker (H), macrophages using CD163 marker (I) and PD-1^+^ lymphocytes (J). Data are shown according to the recurrence status. *N* = 104 patients (52 controls in blue and 52 cases in red) for all analyses, except for PD-1 and PD-L1 markers (*N* = 44, 22 controls, 22 cases). *P*-values are from Wilcoxon test.

**Additional file 5: Fig. S3.** Related to Fig. 2. Increased vascularization is associated with distant relapse. (A) Representative views of CD31 immunostaining in either controls (left) and cases (right) (Scale bar = 100um). (B) Boxplot showing the percentage of CD31+ areas normalized on stroma proportion according to recurrence status and BC molecular subtype (N = 104, 52 controls and 52 cases). *P*-value is from Wilcoxon test.

**Additional file 6: Fig. S4.** Related to Fig. 3. Stroma proportion and content in adipocytes. (A) Percentage of stroma according to recurrence status. The color code in boxplots depicts the BC subtype assessed by Prosigna™ test. Luminal A BC are in light blue (controls, *N* = 37) and light red (cases, *N* = 27). Luminal B BC are in dark blue (controls, *N* = 11) and dark red (cases, *N* = 19). HER2-enriched BC are in light green (controls, N = 2) and dark green (cases, N = 2). The basal like BC is represented in yellow. BC without result for Prosigna™ test are in gray (2 controls and 3 cases). P-value is from Wilcoxon test. (B) Proportion of intratumoral adipocytes according to recurrence status. Controls are in blue and cases are in red. P-value is from Chi2 test. The adiposity of the stroma was evaluated using a semi-quantitative scoring: 0 when the adipose content of the stroma was less than 1% of the surface, 1 between 1 and 10%, 2 between 11 and 30% and 3 if superior to 30%. N = 104, 52 controls and 52 cases. (C) Representative views of CAF marker immunostaining on serial sections from control and case BC patients used for building maps of CAF subsets at cellular scale (shown Fig. 3K) using the decision tree algorithm (shown Fig. 3G).

**Additional file 7: Fig. S5.** Related to Fig. 4. CDH11 expression in tumors. (A) Boxplot showing CDH11 H scores in CAF according to the CAF status (CAF-S1 compared to others) and BC molecular subtype (*N* = 102, 51 controls and 51 cases). H scores are given as a function of percentage of stained CAF multiplied by staining intensity (ranging from 0 to 3). P-value is from Wilcoxon test. The color code depicts the BC subtype assessed by Prosigna™ test. Luminal A BC are in light blue (controls, N = 37) and light red (cases, N = 27). Luminal B BC are in dark blue (controls, N = 10) and dark red (cases, *N* = 18). HER2-enriched BC are in light green (controls, N = 2) and dark green (cases, N = 2). Basal-like BC is in yellow (case, N = 1). BC without result for Prosigna™ test are in gray (2 controls and 3 cases). (B) Same as in (A) for CDH11 H scores in epithelial cancer cells. (C) Representative views of CDH11 immunostaining in epithelial cancer cells (arrows) in controls (left) and cases (right) (Scale bar = 50 μm). (D) Contingency table for the repartition of patients with bone metastases according to the stromal CDH11 H score. *P*-value is from Fisher exact test.

**Additional file 8: Fig. S6.** Related to Fig. 4. Multiple controls upon silencing of CDH11 by siRNA in CAF-S1. (A) Representative western blot (WB) showing CDH11 protein levels (expected molecular weight at 110 kDa) after 3, 5 and 7 days of transfection of CAF-S1 fibroblasts with CDH11-targeted siRNA (siCDH11, pool), and compared to untargeted control siRNA (siCTL). Actin (43 kDa) is used as internal control for protein loading. (B) Barplot showing quantification of CDH11 protein levels (assessed by WB, as shown in A) following transfection by siCDH11 and normalized to siCTL (C) Relative CDH11 mRNA levels assessed by RT-qPCR following 48 and 72 h (H48 and H72) of transfection of CAF-S1 primary fibroblasts with siCDH11 (pool). Data have been normalized on siCTL and cyclophilin mRNA levels for total RNA quantity. (D) Representative WB showing CDH11 protein levels after 3 days of transfection by two siCDH11 (siCDH11#A and siCDH11#B) in two CAF-S1 primary cell lines (CAF-S1 A and CAF-S1 B) and compared to untargeted control siRNA (siCTL). Actin (43 kDa) is used as internal control for protein loading. (E) Barplots showing quantification of CDH11 protein levels (assessed by WB, as shown in D) and normalized to siCTL. (F) Representative WB showing CDH11 protein levels after 3 and 7 days (D3 and D7) of CAF-S1 primary fibroblasts with siCDH11 or siCTL in co-culture conditions in presence of MCF7 cancer cells. (AI 790 Ko). (G-H) Number of CAF-S1 cells transfected with non-targeting siRNA (siCTL) or with CDH11-targeting siRNA (siCDH11), after normalization on carboxylate beads (G) and percentages (%) of alive CAF-S1 cells reported to total number of CAF-S1 (H) upon CDH11 silencing compared to control at 0, 72, 120 and 144 h after transfection. Data are mean ± SEM) (*n* = 4). *P* values are from Mann-Whitney test. (I) Representative image of lower-side Transwell using CAF-S1 into the well. We can observe that migrated CAF-S1 fibroblasts (arrows) are morphologically different from migrated T47D or MCF7 BC cells (shown in Fig. 4H). (J) Images of E-cadherin staining in T47D BC cells in presence of CAF-S1 transfected with control (left) or CDH11-targeting siRNA (right). Scale bars, 20 μm. (K) Quantification of E-cadherin staining per BC cell area (at least three images analyzed per condition). Data are mean ± SEM (*n* = 3). a. u., arbitrary units. P-value from Student t-test.

## Data Availability

All remaining data and materials are available from the authors upon reasonable request.

## Abbreviations

ATCC: American Type Culture Collection
BC: Breast cancer
BCA: Bicinchoninic acid assay
BMI: Body mass index
BSA: Bovine serum albumin
CAF: Cancer-associated fibroblasts
CAF-S1-4: CAF subset 1-4
CD29: Integrin β1
CDH1: E-cadherin
CDH11: Osteoblast cadherin-11
CI95%: 95% confidence interval
CNIL: Commission Nationale de l’informatique et des Libertés
CTL: Control
DC: Dendritic cells
DAPI: 4′,6-Diamidino-2-phenylindole
DC LAMP: Dendritic cell lysosomal associated membrane glycoprotein
DMEM: Dulbecco’s modified Eagle medium
DNA: Deoxyribonucleic acid
ECM: Extracellular matrix
EDTA: Ethylene-diamine-tetra-acetic acid
EGTA: Egtazic acid
EPCAM: Epithelial cell adhesion molecule
ER: Estrogen receptor
FACS: Fluorescence activated cell sorting
FAP: Fibroblast activation protein
FBS: Fetal bovine serum
FOXP3: Forkhead box P3
FSC-A: Forward scatter-A
FSP1/S100-A4: Fibroblast-specific protein 1
H&E: Hematoxylin and eosin
HER2: Human epidermal growth factor receptor-2
HR: Hazard ratio
H score: Histological score
IC: Institut Curie
IHC: Immunohistochemistry
IL17: Interleukin 17
MOPS SDS: Sodium dodecyl sulfate 3-(N-morpholino) propanesulfonic acid
PBS: Phosphate-buffered saline
PCR: Polymerase chain reaction
PD-1: Programmed cell death 1
PD-L1: Programmed death-ligand 1
PDGFRβ: Platelet-derived growth factor receptor-β
PR: Progesterone receptor
qRT-PCR: Quantitative reverse transcription polymerase chain reaction
RNA: Ribonucleic acid
ROR: Risk of recurrence (score defined by the Prosigna™ test)
RPM: Revolutions per minute
RPMI: Roswell park memorial institute medium
RPPA: Reverse phase protein arrays
RT-PCR: Reverse transcription polymerase chain reaction
SEM: Standard errors of mean
siRNA: Small interfering RNA
αSMA: Smooth-muscle α-actin
SSC-A: Sidewards scatter-A
STR: Short tandem repeat
T-Bet: T-box transcription factor
Th1: Type 1 T helper lymphocyte
Th17: Type 17 T helper lymphocyte
TILs: Tumor-infiltrating lymphocytes
TLS: Tertiary lymphoid structures
TME: Tumor micro-environment
T1N0: Tumors with a size ≤ 2 cm and without regional lymph node metastases, as defined by the 8th edition of American Joint Committee on Cancer classification
