# Supplementary material for: A subset of activated fibroblasts is associated with distant relapse in early luminal breast cancer
Source: Breast Cancer Res. 2020 Jul 14;22:76. doi: 10.1186/s13058-020-01311-9 (PMC7362513; doi:10.1186/s13058-020-01311-9)
Supplement: Supplementary file 1 — Additional file 1: Fig. S1. Related to Methods (# Patient cohort) and Table 1. Flow chart for selecting the population of BC patients studied and corresponding survival curves. Flow chart for selecting cases and controls in our cohort (A) and survival analysis of the population of interest (N = 3739) for the recurrence rate (B) and the distant recurrence rate (C). Briefly, the population of interest was female patients with unifocal invasive breast cancer, T1b or T1c N0 M0 (BC smaller or equal to 2 cm, without invaded lymph node and distant metastasis at diagnosis), expressing ER without overexpression of HER2, treated at Institut Curie by at least primary surgery between 2003 and 2010 (N = 3739). Survival curves were constructed according to Kaplan-Meyer method. [file 13058_2020_1311_MOESM1_ESM.pdf]

Additional File 1: Figure S1. Related to Methods (# Patient cohort) and Table 1. Flow chart for selecting the population of BC patients studied and corresponding survival curves. Flow chart for selecting cases and controls in our cohort (A) and survival analysis of the population of interest (N=3739) for the recurrence rate (B) and the distant recurrence rate (C). Briefly, the population of interest was female patients with unifocal invasive breast cancer, T1b or T1c N0 M0 (BC smaller or equal to 2 cm, without invaded lymph node and distant metastasis at diagnosis), expressing ER without overexpression of HER2, treated at Institut Curie by at least primary surgery between 2003 and 2010 (N=3739). Survival curves were constructed according to Kaplan-Meyer method. (AI 505Ko)

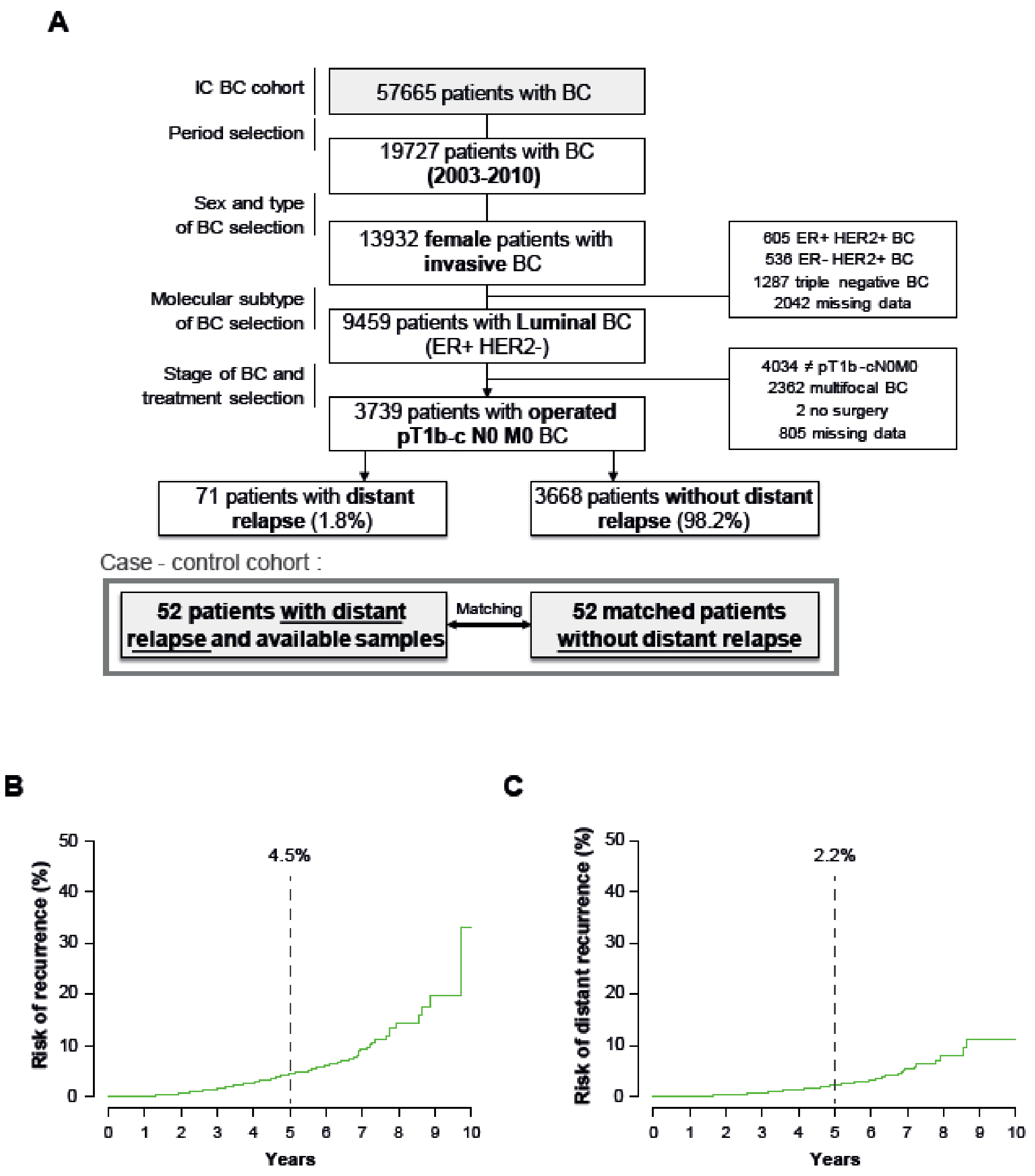
A
IC BC cohort
57665 patients with BC
Period selection
19727 patients with BC (2003-2010)
Sex and type of BC selection
13932 female patients with invasive BC
605 ER+ HER2+ BC
536 ER- HER2+ BC
1287 triple negative BC
2042 missing data
Molecular subtype of BC selection
9459 patients with Luminal BC (ER+ HER2-)
Stage of BC and treatment selection
4034 ≠ pT1b-cN0M0
2362 multifocal BC
2 no surgery
805 missing data
3739 patients with operated pT1b-c N0 M0 BC
71 patients with distant relapse (1.8%)
3668 patients without distant relapse (98.2%)
Case - control cohort :
52 patients with distant relapse and available samples
Matching
52 matched patients without distant relapse
B
4.5%
Risk of recurrence (%)
Years
C
2.2%
Risk of distant recurrence (%)
Years
