# Supplementary material for: A subset of activated fibroblasts is associated with distant relapse in early luminal breast cancer
Source: Breast Cancer Res. 2020 Jul 14;22:76. doi: 10.1186/s13058-020-01311-9 (PMC7362513; doi:10.1186/s13058-020-01311-9)
Supplement: Supplementary file 2 — Additional file 2: Table S1. Related to Methods (# Immunohistochemistry). List of primary antibodies and immunohistochemistry conditions used in the study. [file 13058_2020_1311_MOESM2_ESM.pdf]

Additional File 2: Table S1. Related to Methods (# Immunohistochemistry). List of primary antibodies and immunohistochemistry conditions used in the study. (DOCX 505Ko)

Antibody against	Host, isotype	Reference	Dilution	Antigen retrieval	Secondary kit
**For IHC**					
αSMA	Mouse, IgG1	Dako-M0851	1/350	Citrate pH6	ABC kit
FAP	Rat, IgG2a	Vitatex-MABS1001	1/150	Citrate pH6	ABC kit
CD29	Mouse, IgG1	Abcam-ab3167	1/200	Citrate pH6	ABC kit
FSP1	Rabbit, IgG	Abcam-ab27957	1/450	Citrate pH6	ABC kit
PDGFRβ	Rabbit, IgG	Abcam-ab32570	1/100	Citrate pH6	ABC kit
CDH1	Mouse, IgG1	ThermoFisher 33-4000	1/100	EDTA pH9	Envision
CDH11	Mouse, IgG2b	R&D-MAB1790	1/150	Citrate pH6	ABC kit
CD4	Mouse, IgG1	Novacastra – NCL-CD4-368	1/20	EDTA pH9	Envision
CD8	Mouse, IgG1	Dako-GA62361-2	1/200	EDTA pH9	Envision
FOXP3	Mouse, IgG1	Abcam-ab20034	1/100	EDTA pH9	Envision
CD20	Mouse, IgG1	Dako- GA604	1/100	EDTA pH9	Envision
Tbet	Mouse, IgG1	Santa Cruz sc-21749	1/50	EDTA pH9	Envision
DC Lamp	Rat, IgG2a	Novusbio – DDX0191P	1/100	EDTA pH9	Envision
PD1	Mouse, IgG1	Novusbio – NAT105	1/100	Citrate pH6	Envision
PD-L1	Mouse, IgG1	Dako- M3653	1/25	EDTA pH9	Envision
IL17	Mouse, Polyclonal	R&D – AB10411071	1/100	EDTA pH9	Envision
CD163	Mouse, IgG1	Novacastra – NCL-L-CD163	1/100	EDTA pH9	Envision
CD31	Mouse, IgG1	Dako-M0823	1/100	EDTA pH9	Envision

Abbreviations : αSMA, α-smooth muscle actin; CDH1, E-Cadherin; FAP, Fibroblast Activation Protein α1; IHC, Immunohistochemistry, Integrin β1/CD29, PDGFRβ, Platelet-Derived Growth Factor Receptor-β; S100-A4/FSP1, Fibroblast-Specific Protein 1
