# Supplementary material for: A subset of activated fibroblasts is associated with distant relapse in early luminal breast cancer
Source: Breast Cancer Res. 2020 Jul 14;22:76. doi: 10.1186/s13058-020-01311-9 (PMC7362513; doi:10.1186/s13058-020-01311-9)
Supplement: Supplementary file 3 — Additional file 3: Table S2. Related to Fig. 1. Multivariate analysis using Cox model for risk of distant relapse according to CDH1 (E-Cadherin) expression and proliferation rates. [file 13058_2020_1311_MOESM3_ESM.pdf]

Additional File 3: Table S2. Related to Fig. 1. Multivariate analysis using Cox model for risk of distant relapse according to CDH1 (E-Cadherin) expression and proliferation rates. (DOCX 20Ko)

	Univariate analyses			Multivariate analyses		
	HR	CI95%[]	p value	HR	CI95%[]	p value
				For E-Cadherin and Mitotic Index		
**CDH1**						
Range 250-300	Ref	-	-	Ref	-	-
Per decrease of 50 points	1.21	[1.03-1.44]	0.02	1.20	[1.01-1.43]	0.03
**Mitotic index**						
0-9	Ref	-	-	Ref	-	-
10-19	1.63	[0,88-3,01]	0.11	1.31	[0.68-2.50]	0.40
≥20	1.16	[0,54-2,49]	0.69	1.08	[0.49-2.37]	0.83
				For E-Cadherin and ROR risk category		
**CDH1**						
Range 250-300	Ref	-	-	Ref	-	-
Per decrease of 50 points	1.21	[1.03-1.44]	0.02	1.17	[0.97-1.41]	0.09
**ROR risk category**						
Low risk	Ref	-	-	Ref	-	-
Intermediary risk	1.98	[0.98-4.00]	0.05	1.57	[0.78-3.48]	0.18
High risk	2.45	[1.20-4.98]	0.01	1.89	[0.86-4.12]	0.11

Lobular carcinomas were excluded from analyses using CDH1 (N=12).
