# Supplementary material for: A subset of activated fibroblasts is associated with distant relapse in early luminal breast cancer
Source: Breast Cancer Res. 2020 Jul 14;22:76. doi: 10.1186/s13058-020-01311-9 (PMC7362513; doi:10.1186/s13058-020-01311-9)
Supplement: Supplementary file 4 — Additional file 4: Fig. S2. Related to Fig. 2. Immune profiling in control luminal BC and cases with recurrence. Boxplots showing the count of stained cells per mm2 at the surface of epithelial (left) and stromal (right) compartments for total lymphocytes on HES sections (A), B lymphocytes using CD20 marker (B), CD8+ T lymphocytes (C), CD4+ T lymphocytes (D), FOXP3+ T lymphocytes (E), Th1 T lymphocytes using Tbet marker (F), Th17 T lymphocytes using IL17 marker (G), dendritic cells using DC Lamp marker (H), macrophages using CD163 marker (I) and PD-1+ lymphocytes (J). Data are shown according to the recurrence status. N = 104 patients (52 controls in blue and 52 cases in red) for all analyses, except for PD-1 and PD-L1 markers (N = 44, 22 controls, 22 cases). P-values are from Wilcoxon test. [file 13058_2020_1311_MOESM4_ESM.pdf]

Additional File 4: Figure S2. Related to Fig. 2. Immune profiling in control luminal BC and cases with recurrence.

Boxplots showing the count of stained cells per mm^2 at the surface of epithelial (left) and stromal (right) compartments for total lymphocytes on HES sections (A), B lymphocytes using CD20 marker (B), $CD8^+$ T lymphocytes (C), $CD4^+$ T lymphocytes (D), $FOXP3^+$ T lymphocytes (E), Th1 T lymphocytes using Tbet marker (F), Th17 T lymphocytes using IL17 marker (G), dendritic cells using DC Lamp marker (H), macrophages using CD163 marker (I) and $PD\text{-}1^+$lymphocytes (J). Data are shown according to the recurrence status. N=104 patients (52 controls in blue and 52 cases in red) for all analyses, except for PD-1 and PD-L1 markers (N=44, 22 controls, 22 cases). P-values are from Wilcoxon test. (AI 542 Ko)

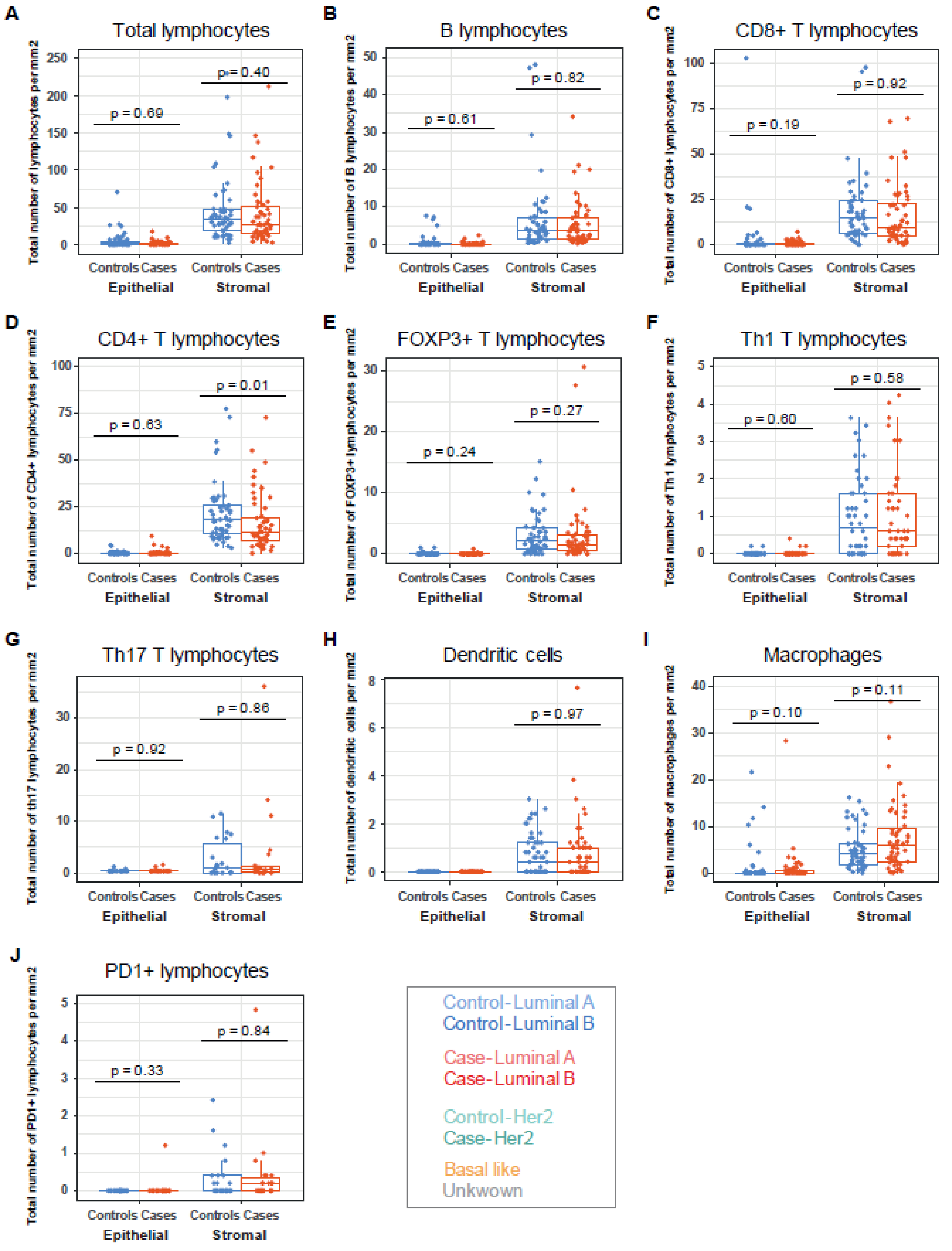
A
Total lymphocytes
Total number of lymphocytes per mm2
p = 0.69
p = 0.40
B
B lymphocytes
Total number of B lymphocytes per mm2
p = 0.61
p = 0.82
C
CD8+ T lymphocytes
Total number of CD8+ lymphocytes per mm2
p = 0.19
p = 0.92
D
CD4+ T lymphocytes
Total number of CD4+ lymphocytes per mm2
p = 0.63
p = 0.01
E
FOXP3+ T lymphocytes
Total number of FOXP3+ lymphocytes per mm2
p = 0.24
p = 0.27
F
Th1 T lymphocytes
Total number of Th1 lymphocytes per mm2
p = 0.60
p = 0.58
G
Th17 T lymphocytes
Total number of th17 lymphocytes per mm2
p = 0.92
p = 0.86
H
Dendritic cells
Total number of dendritic cells per mm2
p = 0.97
I
Macrophages
Total number of macrophages per mm2
p = 0.10
p = 0.11
J
PD1+ lymphocytes
Total number of PD1+ lymphocytes per mm2
p = 0.33
p = 0.84
Controls Cases
Epithelial
Stromal
Control-Luminal A
Control-Luminal B
Case-Luminal A
Case-Luminal B
Control-Her2
Case-Her2
Basal like
Unkwown
