# Supplementary material for: A subset of activated fibroblasts is associated with distant relapse in early luminal breast cancer
Source: Breast Cancer Res. 2020 Jul 14;22:76. doi: 10.1186/s13058-020-01311-9 (PMC7362513; doi:10.1186/s13058-020-01311-9)
Supplement: Supplementary file 5 — Additional file 5: Fig. S3. Related to Fig. 2. Increased vascularization is associated with distant relapse. (A) Representative views of CD31 immunostaining in either controls (left) and cases (right) (Scale bar = 100um). (B) Boxplot showing the percentage of CD31+ areas normalized on stroma proportion according to recurrence status and BC molecular subtype (N = 104, 52 controls and 52 cases). P-value is from Wilcoxon test. [file 13058_2020_1311_MOESM5_ESM.pdf]

Additional File 5: Figure S3. Related to Fig. 2. Increased vascularization is associated with distant relapse.
(A) Representative views of CD31 immunostaining in either controls (left) and cases (right) (Scale bar = 100um). (B) Boxplot showing the percentage of CD31+ areas normalized on stroma proportion according to recurrence status and BC molecular subtype (N=104, 52 controls and 52 cases). P-value is from Wilcoxon test. (AI 7,1 Mo)

A

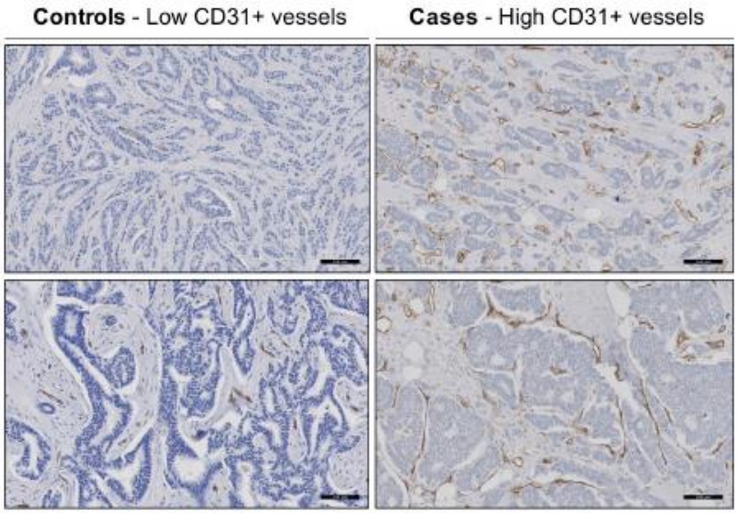
Controls - Low CD31+ vessels
Cases - High CD31+ vessels

B

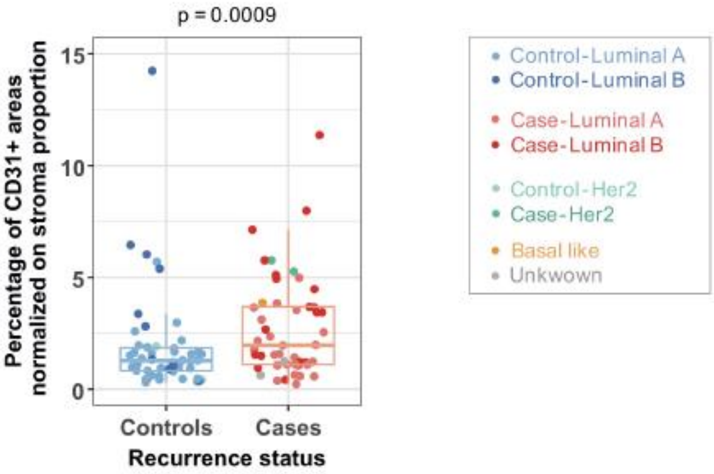
p = 0.0009
Percentage of CD31+ areas normalized on stroma proportion
15
10
5
0
Controls
Cases
Recurrence status
Control-Luminal A
Control-Luminal B
Case-Luminal A
Case-Luminal B
Control-Her2
Case-Her2
Basal like
Unkwown
