# Supplementary material for: A subset of activated fibroblasts is associated with distant relapse in early luminal breast cancer
Source: Breast Cancer Res. 2020 Jul 14;22:76. doi: 10.1186/s13058-020-01311-9 (PMC7362513; doi:10.1186/s13058-020-01311-9)
Supplement: Supplementary file 6 — Additional file 6: Fig. S4. Related to Fig. 3. Stroma proportion and content in adipocytes. (A) Percentage of stroma according to recurrence status. The color code in boxplots depicts the BC subtype assessed by Prosigna™ test. Luminal A BC are in light blue (controls, N = 37) and light red (cases, N = 27). Luminal B BC are in dark blue (controls, N = 11) and dark red (cases, N = 19). HER2-enriched BC are in light green (controls, N = 2) and dark green (cases, N = 2). The basal like BC is represented in yellow. BC without result for Prosigna™ test are in gray (2 controls and 3 cases). P-value is from Wilcoxon test. (B) Proportion of intratumoral adipocytes according to recurrence status. Controls are in blue and cases are in red. P-value is from Chi2 test. The adiposity of the stroma was evaluated using a semi-quantitative scoring: 0 when the adipose content of the stroma was less than 1% of the surface, 1 between 1 and 10%, 2 between 11 and 30% and 3 if superior to 30%. N = 104, 52 controls and 52 cases. (C) Representative views of CAF marker immunostaining on serial sections from control and case BC patients used for building maps of CAF subsets at cellular scale (shown Fig. 3K) using the decision tree algorithm (shown Fig. 3G). [file 13058_2020_1311_MOESM6_ESM.pdf]

Additional File 6: Figure S4. Related to Fig. 3. Stroma proportion and content in adipocytes. (A) Percentage of stroma according to recurrence status. The color code in boxplots depicts the BC subtype assessed by Prosigna$_{TM}$ test. Luminal A BC are in light blue (controls, N=37) and light red (cases, N=27). Luminal B BC are in dark blue (controls, N=11) and dark red (cases, N=19). HER2-enriched BC are in light green (controls, N=2) and dark green (cases, N=2). The basal like BC is represented in yellow. BC without result for Prosigna$_{TM}$ test are in grey (2 controls and 3 cases). P-value is from Wilcoxon test. (B) Proportion of intratumoral adipocytes according to recurrence status. Controls are in blue and cases are in red. P-value is from Chi2 test. The adiposity of the stroma was evaluated using a semi-quantitative scoring: 0 when the adipose content of the stroma was less than 1% of the surface, 1 between 1 and 10%, 2 between 11 and 30% and 3 if superior to 30%. N=104, 52 controls and 52 cases. (C) Representative views of CAF marker immunostaining on serial sections from control and case BC patients used for building maps of CAF subsets at cellular scale (shown Fig. 3K) using the decision tree algorithm (shown Fig. 3G). (AI 354 Ko)

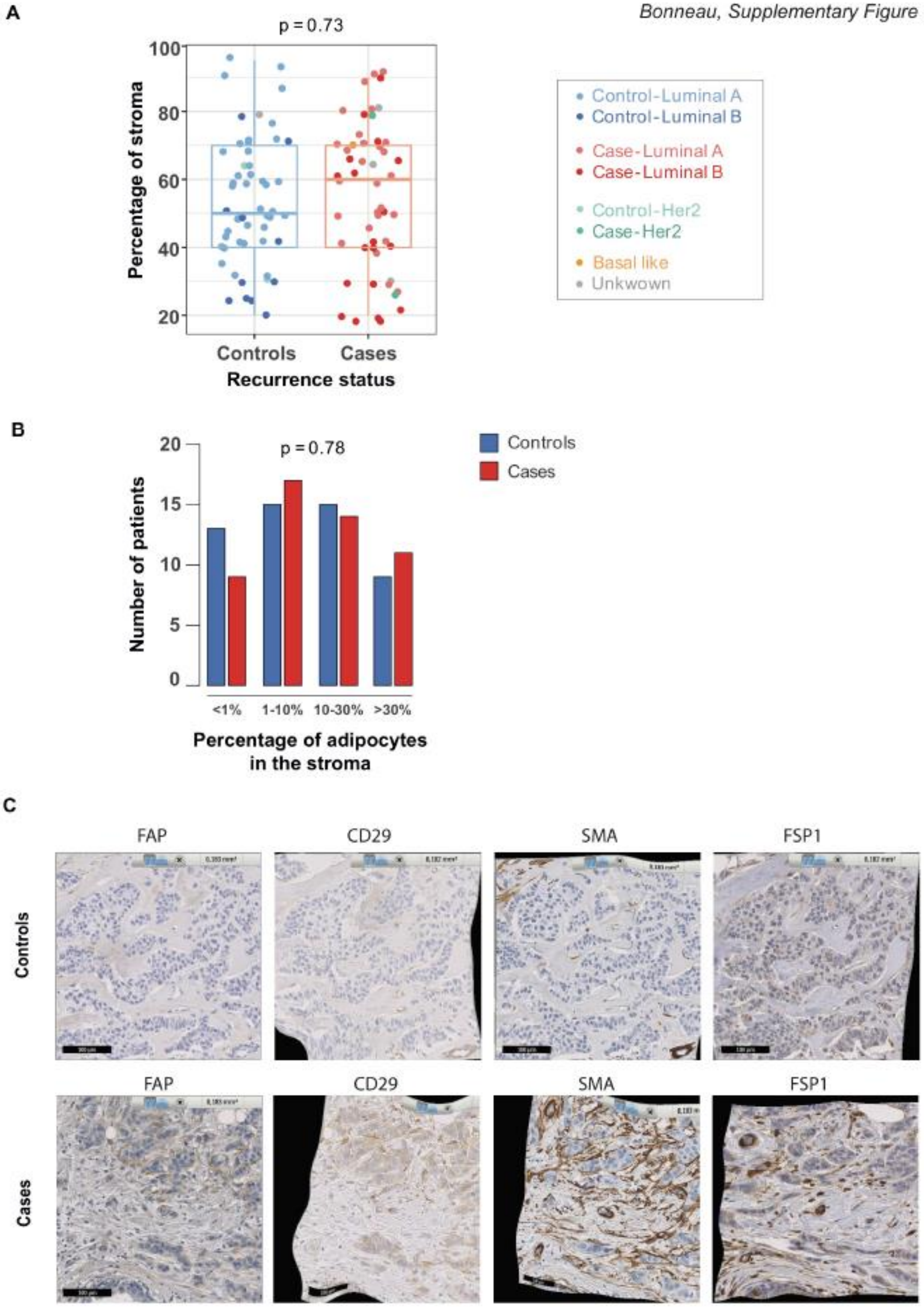
A
p = 0.73
Percentage of stroma
100
80
60
40
20
Controls
Cases
Recurrence status
Control-Luminal A
Control-Luminal B
Case-Luminal A
Case-Luminal B
Control-Her2
Case-Her2
Basal like
Unkwown
B
p = 0.78
Number of patients
20
15
10
5
0
<1%
1-10%
10-30%
>30%
Percentage of adipocytes
in the stroma
Controls
Cases
C
FAP
CD29
SMA
FSP1
Controls
Cases
