# Supplementary material for: A subset of activated fibroblasts is associated with distant relapse in early luminal breast cancer
Source: Breast Cancer Res. 2020 Jul 14;22:76. doi: 10.1186/s13058-020-01311-9 (PMC7362513; doi:10.1186/s13058-020-01311-9)
Supplement: Supplementary file 7 — Additional file 7: Fig. S5. Related to Fig. 4. CDH11 expression in tumors. (A) Boxplot showing CDH11 H scores in CAF according to the CAF status (CAF-S1 compared to others) and BC molecular subtype (N = 102, 51 controls and 51 cases). H scores are given as a function of percentage of stained CAF multiplied by staining intensity (ranging from 0 to 3). P-value is from Wilcoxon test. The color code depicts the BC subtype assessed by Prosigna™ test. Luminal A BC are in light blue (controls, N = 37) and light red (cases, N = 27). Luminal B BC are in dark blue (controls, N = 10) and dark red (cases, N = 18). HER2-enriched BC are in light green (controls, N = 2) and dark green (cases, N = 2). Basal-like BC is in yellow (case, N = 1). BC without result for Prosigna™ test are in gray (2 controls and 3 cases). (B) Same as in (A) for CDH11 H scores in epithelial cancer cells. (C) Representative views of CDH11 immunostaining in epithelial cancer cells (arrows) in controls (left) and cases (right) (Scale bar = 50 μm). (D) Contingency table for the repartition of patients with bone metastases according to the stromal CDH11 H score. P-value is from Fisher exact test. [file 13058_2020_1311_MOESM7_ESM.pdf]

Additional File 7: Figure S5. Related to Fig. 4. CDH11 expression in tumors.
(A) Boxplot showing CDH11 H scores in CAF according to the CAF status (CAF-S1 compared to others) and BC molecular subtype (N=102, 51 controls and 51 cases). H scores are given as a function of percentage of stained CAF multiplied by staining intensity (ranging from 0 to 3). P-value is from Wilcoxon test. The color code depicts the BC subtype assessed by Prosigna™ test. Luminal A BC are in light blue (controls, N=37) and light red (cases, N=27).
Luminal B BC are in dark blue (controls, N=10) and dark red (cases, N=18). HER2-enriched BC are in light green (controls, N=2) and dark green (cases, N=2). Basal-like BC is in yellow (case, N=1). BC without result for Prosigna™ test are in grey (2 controls and 3 cases). (B) Same as in (A) for CDH11 H scores in epithelial cancer cells. (C) Representative views of CDH11 immunostaining in epithelial cancer cells (arrows) in controls (left) and cases (right) (Scale bar = 50 µm). (D) Contingency table for the repartition of patients with bone metastases according to the stromal CDH11 H score. P-value is from Fisher exact test. (AI 3,9 Mo)

Bonneau, Supplementary Figure S5

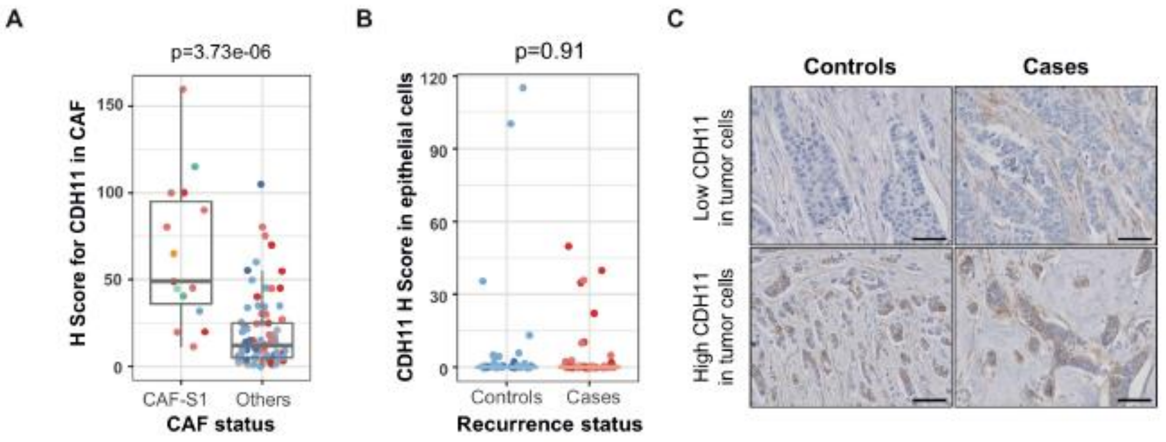

D

p=0.06	Bone metastases	No Bone metastases	Total
CDH11 - Low	15 (29.4 %)	36 (70.6%)	51 (100%)
CDH11 - High	25 (49 %)	26 (51%)	51 (100%)
