# Supplementary material for: A subset of activated fibroblasts is associated with distant relapse in early luminal breast cancer
Source: Breast Cancer Res. 2020 Jul 14;22:76. doi: 10.1186/s13058-020-01311-9 (PMC7362513; doi:10.1186/s13058-020-01311-9)
Supplement: Supplementary file 8 — Additional file 8: Fig. S6. Related to Fig. 4. Multiple controls upon silencing of CDH11 by siRNA in CAF-S1. (A) Representative western blot (WB) showing CDH11 protein levels (expected molecular weight at 110 kDa) after 3, 5 and 7 days of transfection of CAF-S1 fibroblasts with CDH11-targeted siRNA (siCDH11, pool), and compared to untargeted control siRNA (siCTL). Actin (43 kDa) is used as internal control for protein loading. (B) Barplot showing quantification of CDH11 protein levels (assessed by WB, as shown in A) following transfection by siCDH11 and normalized to siCTL (C) Relative CDH11 mRNA levels assessed by RT-qPCR following 48 and 72 h (H48 and H72) of transfection of CAF-S1 primary fibroblasts with siCDH11 (pool). Data have been normalized on siCTL and cyclophilin mRNA levels for total RNA quantity. (D) Representative WB showing CDH11 protein levels after 3 days of transfection by two siCDH11 (siCDH11#A and siCDH11#B) in two CAF-S1 primary cell lines (CAF-S1 A and CAF-S1 B) and compared to untargeted control siRNA (siCTL). Actin (43 kDa) is used as internal control for protein loading. (E) Barplots showing quantification of CDH11 protein levels (assessed by WB, as shown in D) and normalized to siCTL. (F) Representative WB showing CDH11 protein levels after 3 and 7 days (D3 and D7) of CAF-S1 primary fibroblasts with siCDH11 or siCTL in co-culture conditions in presence of MCF7 cancer cells. (AI 790 Ko). (G-H) Number of CAF-S1 cells transfected with non-targeting siRNA (siCTL) or with CDH11-targeting siRNA (siCDH11), after normalization on carboxylate beads (G) and percentages (%) of alive CAF-S1 cells reported to total number of CAF-S1 (H) upon CDH11 silencing compared to control at 0, 72, 120 and 144 h after transfection. Data are mean ± SEM) (n = 4). P values are from Mann-Whitney test. (I) Representative image of lower-side Transwell using CAF-S1 into the well. We can observe that migrated CAF-S1 fibroblasts (arrows) are morphologically different from mi [file 13058_2020_1311_MOESM8_ESM.pdf]

Additional File 8: Figure S6. Related to Fig. 4**.** Multiple controls upon silencing of CDH11 by siRNA in CAF-S1.

(A) Representative western blot (WB) showing CDH11 protein levels (expected molecular weight at 110 kDa) after 3, 5 and 7 days of transfection of CAF-S1 fibroblasts with CDH11-targeted siRNA (siCDH11, pool), and compared to untargeted control siRNA (siCTL). Actin (43 kDa) is used as internal control for protein loading. (B) Barplot showing quantification of CDH11 protein levels (assessed by WB, as shown in A) following transfection by siCDH11 and normalized to siCTL (C) Relative CDH11 mRNA levels assessed by RT-qPCR following 48 and 72 hours (H48 and H72) of transfection of CAF-S1 primary fibroblasts with siCDH11 (pool). Data have been normalized on siCTL and cyclophilin mRNA levels for total RNA quantity. (D) Representative WB showing CDH11 protein levels after 3 days of transfection by two siCDH11 (siCDH11#A and siCDH11#B) in two CAF-S1 primary cell lines (CAF-S1 A and CAF-S1 B) and compared to untargeted control siRNA (siCTL). Actin (43 kDa) is used as internal control for protein loading. (E) Barplots showing quantification of CDH11 protein levels (assessed by WB, as shown in D) and normalized to siCTL. (F) Representative WB showing CDH11 protein levels after 3 and 7 days (D3 and D7) of CAF-S1 primary fibroblasts with siCDH11 or siCTL in co-culture conditions in presence of MCF7 cancer cells. (AI 790 Ko). (G-H) Number of CAFS1 cells transfected with non-targeting siRNA (siCTL) or with CDH11-targeting siRNA (siCDH11), after normalization on carboxylate beads (G) and percentages (%) of alive CAFS1 cells reported to total number of CAF-S1 (H) upon CDH11 silencing compared to control at 0, 72, 120 and 144 hours after transfection. Data are mean ± SEM) (n = 4). P values are from Mann Whitney test. (I) Representative image of lower-side Transwell using CAF-S1 into the well. We can observe that migrated CAF-S1 fibroblasts (arrows) are morphologically different from migrated T47D or MCF7 BC cells (shown in Figure 4H). (J) Images of E-cadherin staining in T47D BC cells in presence of CAF-S1 transfected with control (left) or CDH11-targeting siRNA (right). Scale bars, 20 µm. (K) Quantification of E-cadherin staining per BC cell area (at least three images analyzed per condition). Data are mean ± SEM (n = 3). a. u., arbitrary units. P-value from Student t-test. (AI 16,7 Mo)

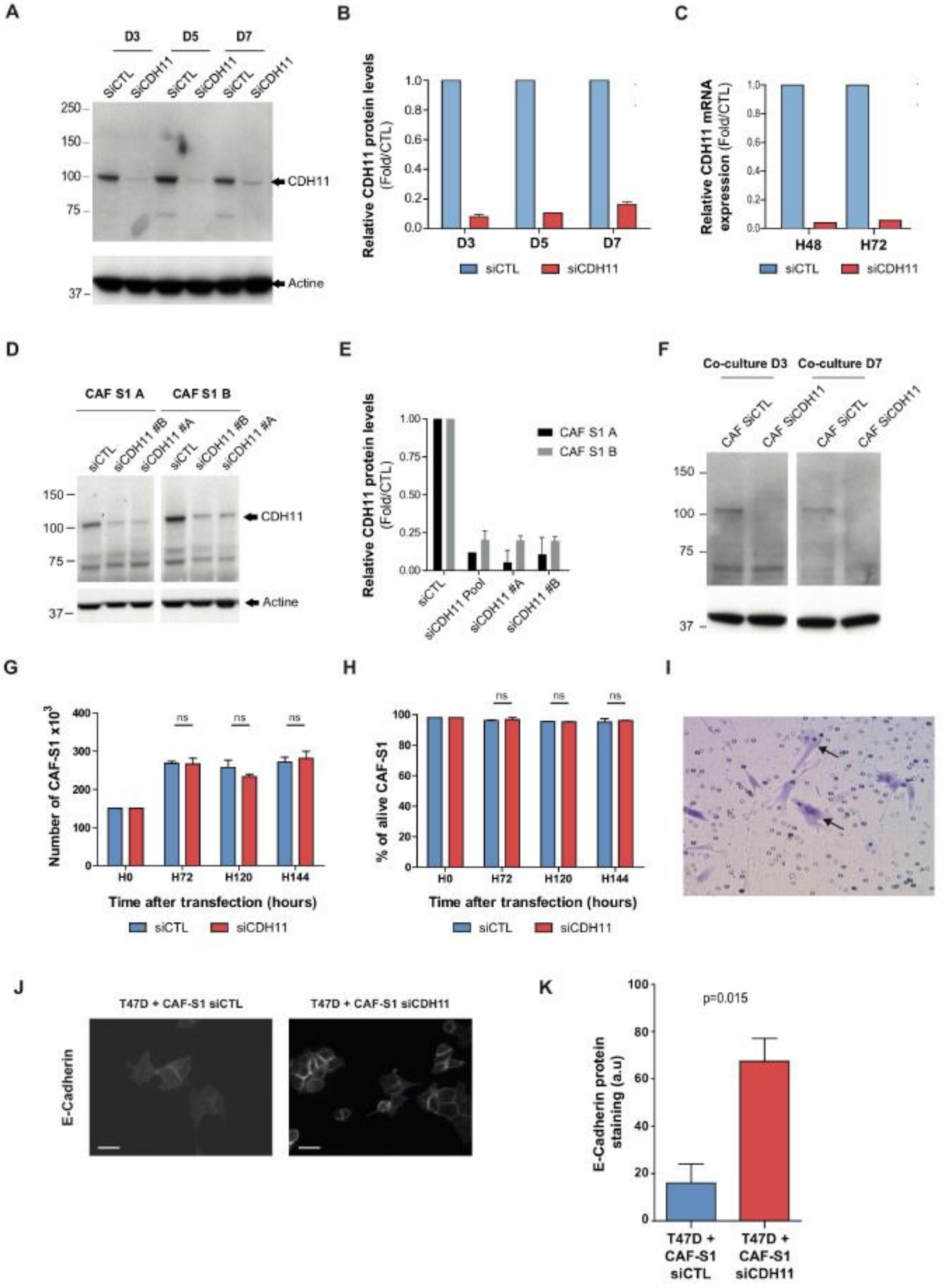
A
D3
D5
D7
SiCTL
SiCDH11
250
150
100
75
37
CDH11
Actine
B
Relative CDH11 protein levels (Fold/CTL)
siCTL
siCDH11
C
Relative CDH11 mRNA expression (Fold/CTL)
H48
H72
D
CAF S1 A
CAF S1 B
siCTL
siCDH11 #B
siCDH11 #A
E
CAF S1 A
CAF S1 B
siCDH11 Pool
F
Co-culture D3
Co-culture D7
CAF SiCTL
CAF SiCDH11
G
Number of CAF-S1 x10^3
ns
H0
H72
H120
H144
Time after transfection (hours)
H
% of alive CAF-S1
I
J
T47D + CAF-S1 siCTL
T47D + CAF-S1 siCDH11
E-Cadherin
K
p=0.015
E-Cadherin protein staining (a.u)
T47D + CAF-S1 siCTL
T47D + CAF-S1 siCDH11
